# Synthesis of a Liquid Lignin-Based Methacrylate Resin and Its Application in 3D Printing without Any Reactive Diluents

**DOI:** 10.1021/acs.biomac.2c01505

**Published:** 2023-03-17

**Authors:** Sarah Keck, Olga Liske, Konstanze Seidler, Bernhard Steyrer, Christian Gorsche, Simone Knaus, Stefan Baudis

**Affiliations:** † Institute of Applied Synthetic Chemistry, Technische Universität Wien, Getreidemarkt 9/163, 1060 Vienna, Austria; ‡ Cubicure GmbH, Gutheil-Schoder-Gasse 17, Tech Park Vienna, 1230 Vienna, Austria

## Abstract

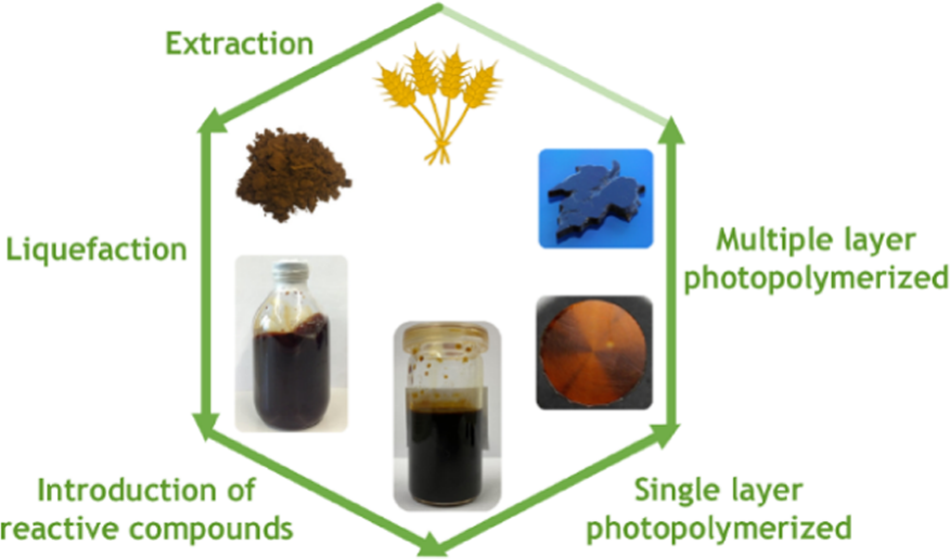

3D printing of bio-based and renewable polymers such as lignin has gained research attention during the last few decades. We report on the synthesis and characterization of a liquid lignin-based photopolymer and its application in additive manufacturing (AM). Wheat straw soda lignin is liquified in an oxyalkylation reaction with propylene oxide under alkaline conditions and modified with methacryloyl chloride to obtain a lignin-based methacrylate resin. Ninety percent of the functional hydroxyl groups are grafted during the synthesis. The photopolymerization efficiency was evaluated by real-time-NIR-photorheology experiments with two different photoinitiators, leading to double bond conversions (DBC) of ≥80%. 3D-printing experiments of the methacrylated lignin were performed with the hot lithography technology. For the first time, a light-curable lignin derivative with a lignin content of over 30% was successfully 3D printed via vat photopolymerization without any reactive diluents, which is a significant improvement over current state-of-the-art solutions. This outstanding result is a motivating proof of concept and a promising starting point for the in-depth evaluation of bio-based precursors as an alternative to nonrenewable derivatives for 3D printing.

## Introduction

Photopolymerization is a well-established technology and finds application in fields such as electronics,^1^ optics,^2^ biomedical engineering,^3^ dental materials,^4^ and 3D printing.^5,6^ 3D printing, especially lithography-based additive manufacturing technologies (L-AMTs), is a powerful tool for manufacturing complex parts with exceptional surface quality. However, commercially available materials are predominantly based on petrochemical products leading to products with very low sustainability. The interest in photopolymerization using bio-based compounds from renewable resources has risen during the last decade, where lignin is an attractive candidate. With an estimated resource of 300 billion metric tons, lignin is the second most abundant biopolymer on earth.^7^ Depending on the isolation methods, sulfur-containing (Kraft lignins and lignosulfonates) or sulfur-free (soda and organosolv lignins) lignins with different structural parameters and properties are obtained.^8^ Although most of the obtained lignin from the pulp and paper industry is burned to generate energy, there have been approaches to developing increased value applications with lignins.^9^ There, lignin is used either directly or chemically modified as a raw material for several bio-based polymers such as polyesters,^10^ phenol-formaldehyde resins,^11^ polyurethanes,^12−14^ copolymers,^15^ blends,^16^ or UV coatings.^17,18^ In the literature, many studies focused on chemical modification of the functional hydroxyl groups to synthesize bio-based polyols.^19^ Propylene oxide is widely used by applying ring-opening polymerization with epoxides as a synthetic approach. Wu and Glasser^20,21^ first studied oxyalkylation reactions of Kraft and sulfite (lignosulfonate) lignins. Nadji and Cateto^12,13^ performed several oxypropylation experiments on lignins that were used as precursors for polyurethanes.

Nevertheless, different lignin systems are already implemented in the field of 3D printing, where lignin acts as a filler material. In the category of material extrusion-based techniques (MEX), fused filament fabrication (FFF), also known under the trademarked name fused deposition modeling (FDM), and direct ink writing (DIW), thermoplastic filaments are processed in the case of DIW pastes or solution-based hydrogels as well. For FFF, lignin is used as a compound with acrylonitrile butadiene styrene (ABS),^22^ polylactide acid (PLA),^23,24^ or polyhydroxybutyrate (PHB).^25^ In DIW, different types of lignins are blended with hydroxypropylcellulose,^26,27^ cellulose nanofibers, and alginate,^28^ or an acrylate-containing soft triblock copolymer Pluronic F127.^15^ Vat photopolymerization (VPP), which includes many of the L-AMTs such as stereolithography (SLA), digital light processing (DLP), or LCD technology, is based on a light-induced polymerization of low viscosity and photoactive resin with a photoinitiator.^29^ (Meth)acrylic- and epoxy-based monomers are usually used for this method.^30^ In 2019, Zhang^31^ and Ibrahim^32^ reported on the incorporation of small amounts (up to 3 wt %) of Kraft and organosolv lignin into commercially available resins. Major enhancements in lignin incorporation leading to improved performance properties were achieved using chemically modified lignin. Sutton and co-workers modified organosolv lignin with methacrylic anhydride, and it was possible to incorporate up to 15 wt % of the modified lignin into an SLA resin.^33^ The latest results focused on improved UV curing of organosolv lignin-containing photopolymers. Lignin reduction and acylation were used as chemical modification techniques to reduce the UV absorption by lignin, and the resulting mechanical and cure properties are discussed.^34^ Furthermore, recent developments showed that chemical-modified lignin can be used as a photoinitiator (PI) as well.^35−37^ The current state-of-the-art of 3D printing of lignin, including challenges and opportunities, is well-summarized by Ebers et al.^30^

**Figure 1:**
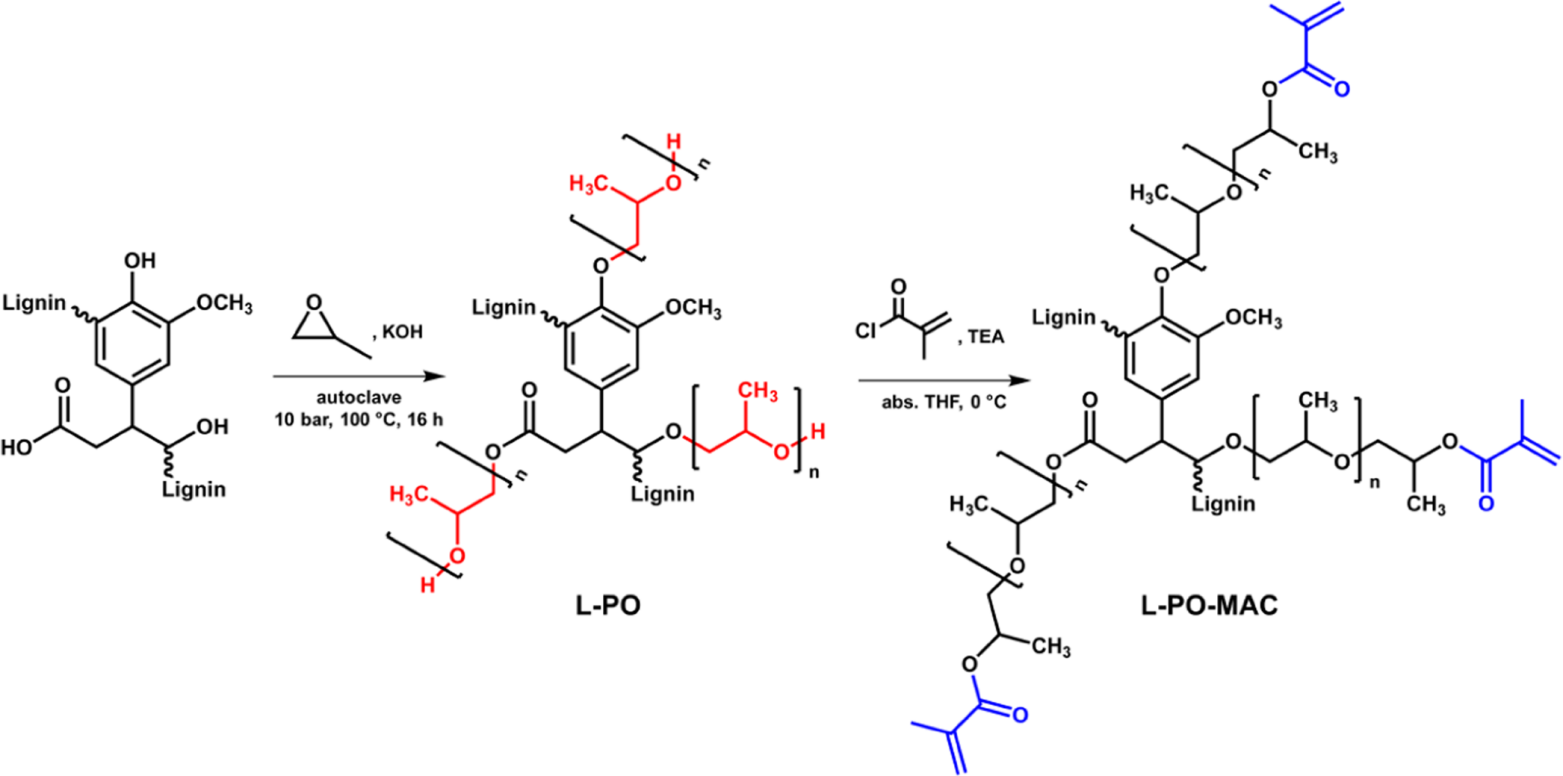
Synthetic pathway for modification of wheat straw lignin to obtain the photopolymerizable macromonomer.

Herein, we report a 2-step synthetic pathway for developing a liquid lignin-based methacrylate with a lignin content between 26 and 39% that can be photopolymerized as an additive with other (meth)acrylic resins or as a pure component without any reactive diluents. First, lignopolyol (L-PO) is synthesized via oxyalkylation of wheat straw soda lignin with propylene oxide (PO). Second, lignopolyol is converted into a macromonomer with methacryloyl chloride (Figure 1). The efficiency in photopolymerization is evaluated by real-time-NIR-photorheology experiments,^38^ followed by 3D-printing experiments via hot lithography, which is a unique SLA technology enabling the processing of viscous resins (up to 20 Pa·s) at elevated processing temperatures (up to 120 °C).^39^ There have been no reports in the literature using a liquid lignin methacrylate (L-PO-MAC) photopolymer only with a photoinitiator for application in L-AMTs.

## Experimental Section

### Materials

Wheat straw soda lignin Protobind1000 (PB1000) (Greenvalue SA, Orbe, Switzerland) was used for all experiments. (±)-Propylene oxide (99%, Sigma-Aldrich) was used as an oxyalkylation agent. Liquid–liquid extraction of lignopolyol was performed with acetonitrile (ACN, 99.9%, VWR) and *n*-hexane (97%, VWR). Methacryloyl chloride (MAC, Alfa Aesar) was distilled before usage to remove cyclic dimers. Triethylamine (TEA, 99%, Sigma-Aldrich) was used as a catalyst and acid scavenger. For quantitative ^31^P-NMR spectroscopy, lignin samples were derivatized with 2-chloro-4,4,5,5-tetramethyl-1,3,2-dioxaphospholane (TMDP, Sigma-Aldrich). Cyclohexanol (Sigma-Aldrich) was used as an internal standard for quantification. Bis(4-methoxybenzoyl) diethylgermanium (Ivocerin, Ivoclar Vivadent) and bis(2,4,6-trimethylbenzoyl)-phenylphosphine oxide (Irgacure 819, Ciba Specialty Chemicals) were used as photoinitiators for photopolymerization experiments, respectively.

### Lignin Modification

#### Synthesis of L-PO

Oxyalkylation was performed according to Wallisch et al.^40^ in a Berghof autoclave BR750. Twenty-five grams (1 equiv) of wheat straw soda lignin (OH number determined by ^31^P-NMR spectroscopy) and finely grounded KOH (2.6 mmol·mol^–1^ PO + 1 equiv for neutralization of carboxylic OH) were weighed into the PTFE insert with a mechanical stirrer. Finally, 10 equiv of PO was added via a syringe, and the autoclave was closed. After purging with gaseous nitrogen, a pressure of 10 bar N_2_ was applied. The oxyalkylation reaction was stirred at 300 rpm at 100 °C for 16 h. After cooling down to room temperature, (RT) the pressure was released completely. The dark brownish and viscous content was dissolved in acetonitrile and centrifuged (15 min, 10 °C, 5000 rpm) to get rid of insoluble components. The centrifuged mixture was extracted through liquid–liquid extraction with an extractor for specifically light solvents to remove the PO homopolymer. The extraction took place by refluxing *n*-hexane for at least 48 h. When cooling down to RT, the phases were separated (dark brown ACN and yellow *n*-hexane solution) and evaporated to dryness at a membrane pump vacuum and then further dried with a high vacuum pump by water bath heating.

#### Synthesis of L-PO-MAC

The synthesis was performed similarly to the work of Hajirahimkhan and co-workers.^17,18^ One equivalent of L-PO (OH number determined by ^31^P-NMR spectroscopy) was transferred to a three-necked flask and dissolved in dry THF (40 mL per 5 g of lignin). The brown solution was purged with argon, and TEA (3 equiv) was added. Afterward, the solution was cooled to 0 °C. Then, 1.5 equiv of freshly distilled methacryloyl chloride was added dropwise to the brown solution within 1 h. The mixture was stirred under an inert atmosphere at RT overnight and precipitated in a 10-fold amount of slightly acidic water (pH 3). The supernatant was decanted, and the brown residue was dissolved in a small amount of DCM because it dissolved lignin and was easily removed by evaporation. Both fractions were centrifuged (5 min, 10 °C, 5000 rpm). The dark brown DCM solution was extracted in a separating funnel with hydrochloric acid (1 M), sodium hydroxide (1 M), and water several times to remove excessive TEA and MAC and for neutralization of the extract. The organic phase was dried with anhydrous sodium sulfate and filtered, and the filtrate was dried under reduced pressure for 48 h. The theoretical yield of L-PO-MAC samples and the conversion of OH groups into reactive double bonds can be calculated with eqs 1 and 2.
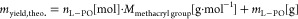
1
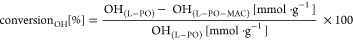
2

#### L-PO-MAC Master Mixture

Eighteen weight percent L-PO-MAC 1, 36 wt % L-PO-MAC 2, and 46 wt % L-PO-MAC 3 were dissolved in DCM and dried again under reduced pressure to generate a master mixture of 76 g for 3D-printing experiments.

### Lignin Characterization

#### ^13^C- and 2D-HSCQ-NMR Spectroscopy

One hundred milligrams of PB1000 was dissolved in 0.6 μL of DMSO-*d*_6_, and 50 μL of Cr(acac)_3_ in DMSO-*d*_6_ (concentration: ∼50 mg·mL^–1^) was added as a relaxation agent. The sample was homogenized with a vortex and measured on a Bruker Avance 600 NMR spectrometer.^13^C-NMR spectra were recorded with an *igated* mode (15360 scans, 0.9 s acquisition time, 2.0 s relaxation delay). For 2D-HSQC-NMR spectra (1024 × 128 matrix), the Bruker standard pulse sequence “hsqcetgpsisp2” was used. Mestrenova version 12.0.0-20080 was used for the analysis (see the Supporting Information) of the NMR spectra.

#### ^31^P-NMR Spectroscopy

Approximately thirty milligrams of lignin sample was dissolved in 200 μL of a dimethylformamide (DMF)/pyridine mixture (1:1, v/v) and shaken with a vortex mixer. Then, 100 μL of a cyclohexanol/Cr(acac)_3_ mixture in pyridine (concentration: ∼40 mg·mL^–1^ cyclohexanol and ∼5.0 mg·mL^–1^ Cr(acac)_3_) was added to the lignin sample and homogenized with a vortex. Separately, 400 μL of deuterated chloroform (CDCl_3_) and 100 μL of TMDP were mixed and applied to the lignin sample solution. After a short mixing time, the ^31^P-NMR spectrum was measured immediately. ^31^P-NMR-spectra were recorded on a Bruker Avance 400 NMR spectrometer with an igated mode (256 scans, 25 s relaxation time, duration of 1 h 50 min). Using a 600 MHz spectrometer, the measuring time was reduced to 128 scans. All ^31^P-NMR-spectra were analyzed with Mestrenova version 12.0.0-2008. The signal of the internal standard is set to 1, followed by integration of the 3 regions and calculation of the OH number according to eq 3. Integration areas (Table S3) and further Information are given in the Supporting Information.
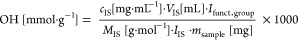
3

#### ^1^H-NMR Spectroscopy

For acetylation of L-PO, 1 g of L-PO was dissolved in 8.1 mL of pyridine (0.1 mol, 1 equiv) and 5 mL of acetic anhydride (0.1 mol, 1.6 equiv) and stirred for 24 h at RT. After 24 h, the brownish liquid was cooled with an ice bath, and 50 mL of ethanol was added. The solution was stirred for 30 min, and the solvent was evaporated to dryness with a membrane pump vacuum afterward. The procedure was repeated 3 times. The residue was extracted with a dichloromethane/water mixture, and the organic phase was dried with sodium sulfate. After filtration, the product was dried with a membrane pump vacuum. ^1^H-NMR spectra were measured in 0.6 μL of CDCl_3_ or DMSO-*d*_6_ with a Bruker Avance 400 or Bruker Avance 600 MHz spectrometer (16 scans). Mestrenova version 12.0.0-20080 was used for the analysis of the NMR spectra.

#### FTIR Spectroscopy

Infrared transmission measurements were taken via FTIR Spectrometer V 10.01.00.0030 from PerkinElmer in attenuated total reflectance (ATR) mode. The spectra were measured in the range between 600 and 4000 cm^–1^. Each measurement consists of 32 scans with a resolution of 4 cm^–1^, and the measurements were taken at RT. Before starting the measurement, a background spectrum was recorded. Received spectra were analyzed with PerkinElmer Spectrum version 10.03.07 software.

#### Viscosity

The viscosity of propoxylated (methacrylated) lignins was determined with an Anton Paar MCR 300 rheometer with a cone-plate order CP25-1-SN40166 and a measuring distance of 0.048 mm. The shear rate was 100 s^–1^. Measurements were taken at 25 °C, and the temperature was adjusted by means of Peltier elements. For every experiment, at least triplicates were measured. The results were analyzed with RheoPlus/32 V3.62 software.

#### Elemental Analysis

The carbon, hydrogen, nitrogen, oxygen, and sulfur contents were determined with an EA 3000 CHNS-O Elemental Analyzer from Eurovector.

#### Size Exclusion Chromatography

Gel permeation chromatography (GPC) measurements were performed on a Malvern VISCOTEK TDA system using UV detection. Samples were prepared as syringe-filtered 3 mg·mL^–1^ THF-solutions spiked with 0.5 mg·mL^–1^ butylhydroxytoluol (BHT) as a flow marker. Separation was conducted through three consecutive PSS SDV columns (100, 1000, and 100,000 Å) using THF as a solvent at a flow rate of 0.8 mL·min^–1^. Conventional calibration was done with polystyrene standards between 479 and 488,000 g·mol^–1^. The results were analyzed with OmniSEC version 5.10.461 software by Malvern. PB1000 and L-PO samples were acetylated as described in the ^1^H-NMR spectroscopy paragraph before the measurement.

### Photopolymerization of Modified Lignin

#### Formulations

For real-time-NIR-photorheology measurements, formulations were prepared freshly and stored under the exclusion of light. Monomer (L-PO-MAC) and PI (Ivocerin or BAPO) were weighed in a brown glass vial and mixed with a vortex mixer. Afterward, the vial was submerged in an ultrasonic bath for 30 min.

#### Real-Time-NIR-Photorheology

The experimental setup was used as described in the literature.^38^ Samples were measured with an Anton Paar MCR302 WESP device with a P-PTD 200/GL Peltier glass plate and a PP25 measuring system and a gap size of 50 μm. Each formulation was sheared with a frequency of 1 Hz (shear strain 1%) and irradiated with an OmniCure 2000 UV-lamp (wavelength 320–500 nm) with an intensity of 50 mW·cm^–2^ for a period of 300 s at RT. The OmniCure 2000 Hg lamp was calibrated once before the measurements using an Ocean Optics device. A set of IR spectra was recorded during irradiation (time interval ∼0.26 s) with a Bruker Vertex 80 FTIR spectrometer using OPUS V7.0.129 software. For reproducibility, at least triplicate measurements were performed for all formulations. Rheology data was analyzed by RheoCompass V1.24.510 software from Anton Paar.

### 3D Printing and Characterization of Printed Specimen

#### Formulations

For 3D printing, the formulations were prepared via speed mixing with a DAC 150.1 FVZ-K from Hauschild. The lignin resin and 3 wt % PI were weighed in a speed mixing cup and placed in an oven at 75 °C for 1 h. Afterward, the 75 °C resin was mixed for 2 × 30 s at 1800 and 2300 rpm.

#### 3D Printing

3D printing was conducted on a hot lithography printer of the Caligma 200 series of Cubicure GmbH. Samples were printed at 40 °C and cured by a 405 nm laser source with an energy density of 3167 mJ·cm^–2^.

#### Dynamic Mechanical Thermal Analysis (DMTA)

DMTA measurements were performed with an Anton Paar MCR301 device with a CTC 450 oven. The specimens were measured in torsion mode with a frequency of 1 Hz and a strain of 0.1%. The temperature was increased from −100 to 200 °C with a heating rate of 2 °C min^–1^. The glass transition temperature was defined as the temperature at the maximum dissipation factor (tan δ). DMTA data was analyzed by RheoCompass V1.24.510 software from Anton Paar.

#### Tensile Tests

According to ISO 527-2, tensile tests of the 3D-printed specimen (type 5B) were measured on a Zwick ProLine Z010. A preload of 0.1 MPa was applied, and Young’s modulus was evaluated between 0.05 and 0.25% elongation (regression) with a speed of 0.25 mm·min^–1^. The test speed was 5 mm·min^–1^, and the strain was measured via traverse using the clamping distance. At least 5 specimens were tested.

#### Scanning Electron Microscopy (SEM)

SEM measurements were performed on an FEI Philips XL30 scanning electron microscope. Test samples were coated with a thin conducting gold layer with an Agar Sputter Coater B7340 before the measurement.

#### Digital Microscopy

The 3D-printed specimen was analyzed with a Keyence VHX6000 digital microscope.

## Results and Discussion

### Modification and Characterization of Lignin

**Figure 2:**
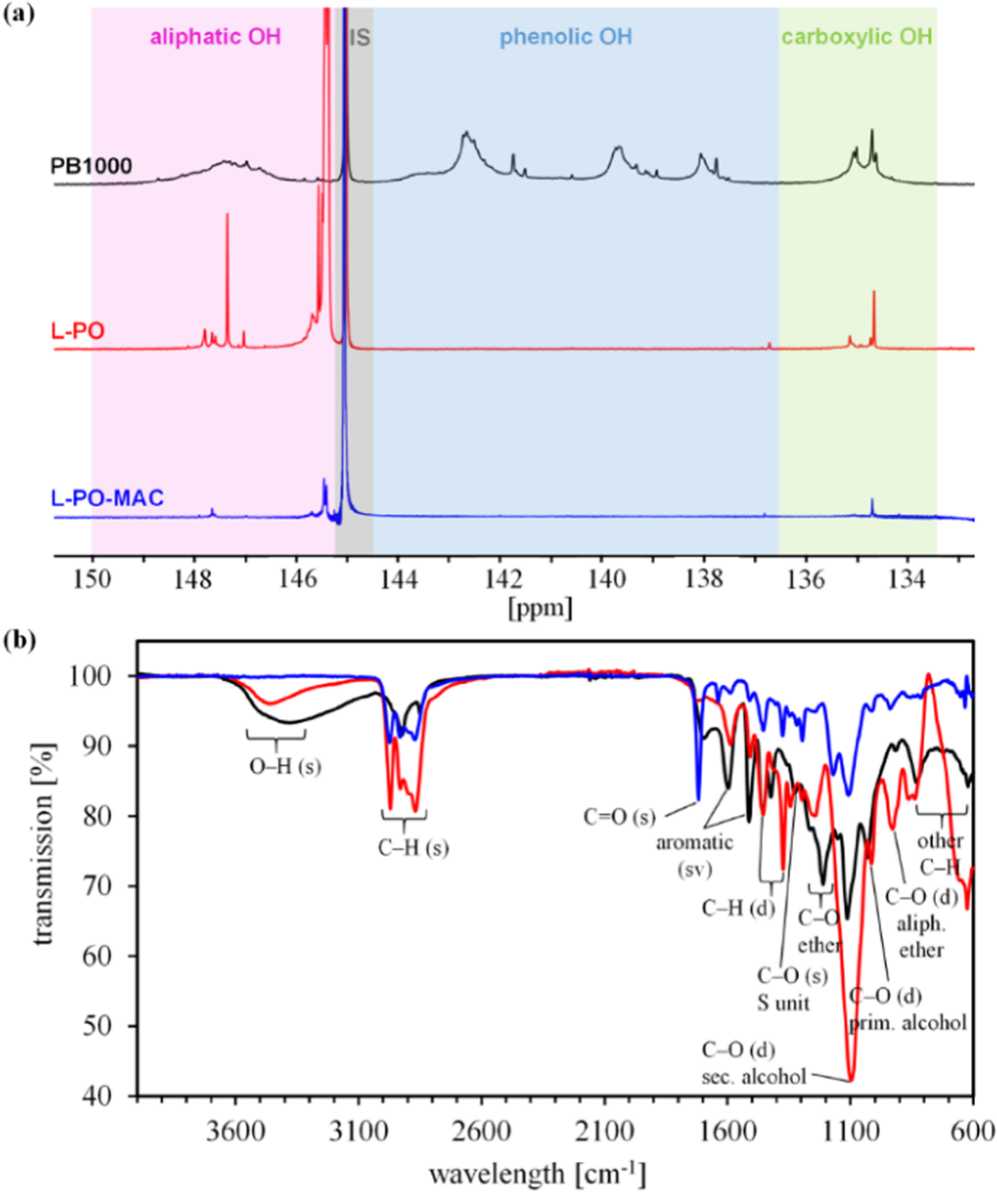
Spectroscopy results of PB1000, propoxylated lignin L-PO, and methacrylated propoxylated lignin L-PO-MAC. (a) Quantitative ^31^P-NMR spectra. The signal of the internal standard cyclohexanol (145 ppm) is set to 1, and spectra are integrated with the following range: carboxylic OH (133.5–136.5 ppm), phenolic OH (136.5–144.5 ppm), and aliphatic OH (145.2–150.0 ppm). A more detailed assignment is given in Figure S4 and Table S3. (b) ATIR spectra with the assignment of the characteristic peaks.

Wheat straw soda lignin Protobind1000 is used as a raw material. Soda lignin was chosen since they are sulfur-free and obtained with a relatively low sugar and ash content.^41−44^ It is known from the literature that PB1000 contains 2–4% polysaccharides, mainly xylose as a hemicellulose derivative.^41,44^ Elemental analysis of PB1000 (C: 62.4%, H: 6.14%, N: 1.16%, O: 28.3%, S: 0.36%) shows comparable results known from the literature.^41,45^ Technical soda lignins have a number-average molecular weight (*M*_n_) of 800–3000 g·mol^–1^ and a polydispersity of 2.5–3.5.^8^ The number-average molecular weight (*M*_n_), the weight-average molecular weight (*M*_w_), and the polydispersity index (*M*_w_/*M*_n_) of PB1000 were determined via GPC measurements to be 900, 2300, and 2.5 g·mol^–1^, respectively. PB1000 was characterized via spectroscopic methods before usage. The Fourier transform infrared (FTIR) spectrum (Figure 2b) shows the typical broad OH peak at 3371 cm^–1^ and C–H stretching at 2920 and 2850 cm^–1^. Aromatic ring vibration bands of PB1000 can be observed at 1597 and 1512 cm^–1^. C–H deformation peaks appear at 1459 and 1423 cm^–1^. C–O stretching of the syringyl unit appears at 1327 cm^–1^. The peak at 1212 cm^–1^ can be assigned to C–O–C soft segments. C–H stretching peaks of polysaccharides are observed at 1113 and 1031 cm^–1^. The peaks at 832 and 622 cm^–1^ reflect other C–H groups. ^13^C- and 2D-HSQC-NMR spectra were recorded to get better information about the structure and linkages of PB1000. NMR signals were assigned according to the published literature (del Río,^46^ Sun,^43^ Wen,^47,48^ and Yuan,^49^). The 2D-HSQC-NMR spectrum can be divided into two regions. In the aromatic region, the corresponding signals of the guaiacyl (G), *p*-hydroxyphenyl (H), and syringyl (S) lignin units are observed, as well as *p*-coumarate (PCA) and ferulate (FA) substructures, which are present in lignins obtained from grasses.^46,47^ The side-chain region gives information about interunit linkages in lignin and possible lignin–carbohydrate complex (LCC) linkages. From the major lignin linkages (β*-*O-4′, β-β′, β-5′, β-1′, etc.),^49^ only β-O-4′ aryl ether and β-β′ resinol substructures were observed in the HSCQ spectrum. Since PB1000 contains xylose, β-D-xylopyranoside cross signals are obtained as well. The main LCC structures are phenyl glycoside bonds, esters, and benzyl ethers (BE). There is no evidence of phenyl glycoside or esters cross signals in the HSQC spectrum; only a weak signal of benzyl ether linkages was detected. The NMR spectra and signal assignments of the spectra are given in the Supporting Information (Figures S1 and S2 and Tables S1 and S2).

In this work, the OH number of the used lignins is a critical parameter for several calculations, including synthetic approaches, lignin content, yields, and conversion of OH groups into photopolymerizable double bonds. The OH number is determined by quantitative ^31^P-NMR spectroscopy.^50−53^ Therefore, lignin samples are derivatized with 2-chloro-4,4,5,5-tetramethyl-1,3,2-dioxaphospholane (TMDP), and cyclohexanol is used as the internal standard (IS) (see Figure S3). The integration areas, based on the work of Ahvazi et al.,^45^ are listed in Table S3 in the Supporting Information. The resulting ^31^P-NMR spectra (Figure 2a) can be divided into 3 regions for the different functional OH groups of lignin: carboxylic, phenolic, and aliphatic OH groups. PB1000 has a total OH number of 7.58 mmol·g^–1^ (aliphatic OH: 1.88 mmol·g^–1^; phenolic OH: 4.55 mmol·g^–1^, and carboxylic OH: 1.15 mmol·g^–1^), which is used for the calculation of the first synthetic step. The phenolic OH region can be subdivided into condensed and uncondensed (S–OH, G–OH, and H–OH) phenolic OH groups. A detailed.^31^P-NMR spectrum of PB1000 is shown in Figure S4. All 3 monolignols are present, and the S/G/H ratio was calculated to be 45/37/18. The amount of condensed/uncondensed phenolic OH is 19/81%.

PB1000 (1 equiv with respect to hydroxy groups) is liquefied through an oxyalkylation reaction with propylene oxide (10 equiv) under alkaline conditions (KOH, 0.2 equiv) in a solvent-free procedure (Figure 1). The potassium hydroxide amount includes the amount to neutralize the carboxylic OH groups as well as excess to establish the necessary alkaline conditions. Oxypropylation is performed in a Berghof autoclave, according to the process patented by Wallisch et al. in 2017.^40^ Three fractions, namely, a lignin copolymer (lignopolyol), a PO homopolymer, and an insoluble part, are obtained. The fractions are separated as described in the literature.^12,20,40^ After oxypropylation, acetonitrile (ACN) is added to the dark brownish mixture, and insoluble components are separated by centrifugation. Liquid–liquid extraction with *n*-hexane is performed to remove the PO homopolymer.^12,20,40^ Instead of *n*-hexane, diethyl ether or tetrachloromethane could also be used, but both solvents are unsuitable for quantitative separation since they can extract parts of the lignopolyol.^19,54^ For some applications, e.g., polyurethanes.^55^ it is unnecessary to remove the PO homopolymer fraction so that the liquid–liquid extraction could be omitted. However, the extraction step is indispensable to keep the lignin content as high as possible in the products. The fractions of unreacted lignin (brown solid), PO homopolymer (yellow), and brown propoxylated lignin (L-PO) are dried, and the weight is determined gravimetrically. For a better understanding, a schematic of the synthetic pathway is given in Figure S5. The mass percentage (m/m) of a fraction (yield by weight) can be calculated by dividing the mass of an obtained fraction by the sum of all products. L-PO is characterized by NMR- and FTIR spectroscopy, followed by the determination of the viscosity via rheology. Since sample amounts of around 80 g are required for 3D-printing experiments and the autoclave size limits the yield of L-PO, the oxyalkylation step was repeated several times also to study the reproducibility of the process. The results of L-PO 1–4 are summarized in Table 1. For the synthesis of L-PO 1, a PB1000 lignin with an OH number of 6.57 mmol·g^–1^ was used. Due to the larger availability and better yield of L-PO, the previously described PB1000 (7.58 mmol·g^–1^) batch was used for the L-PO 2–4 syntheses. The FTIR spectrum (Figure 2b) of L-PO products, in general, shows a decreased OH band at 3443 cm^–1^ and the formation of a new peak at 1371 cm^–1^, which can be assigned to the CH_3_ of the propyl group. An increase of the C–H stretching peaks at 2967 and 2864 cm^–1^ and the C–O peak of secondary alcohols at 1090 cm^–1^ can be observed. After oxyalkylation, most of the OH groups are grafted with poly(propylene glycol) (PPG) chains, which is shown in the quantitative ^31^P-NMR spectrum (Figure 2a) by the formation of an oxypropyl peak at 145.5 ppm. Dividing the sum of all OH numbers of unreacted OH groups by the starting OH number of PB1000, we can conclude that only 3–4% of OH groups in L-PO 1–4 are not grafted with PO, and the reaction runs near to quantitative. The OH number decreases with the oxyalkylation process. Since the OH number is referenced to the sample mass, a decreasing OH number correlates with an increase in grafted chain length and yield of L-PO. The lignin content (Table 1, eq, see the Supporting Information) in L-PO samples can be calc ulated via ^31^P-NMR spectroscopy by dividing the OH number of L-PO by the OH number of PB1000. The neat lignin content after oxyalkylation is in a range between 29 and 41%. The grafted PPG chain length can be determined by acetylation of L-PO with acetic anhydride and pyridine at room temperature and subsequent analysis of the acetylated lignopolyol via ^1^H-NMR spectroscopy (more details are available in the Supporting Information). The ^1^H-NMR spectrum (see Figure S6) shows four peaks at 5 ppm (a CH group at the chain end), 4.0–3.1 ppm (a CH_2_–CH group of the PPG ether backbone), 2.0 ppm (CH_3_ of the acetate group), and 1.4–0.8 ppm (CH_3_ of a propyl group of PPG ether), respectively. By dividing the respective integral of the CH_3_ group of the PPG ether by the integral of the CH_3_ of the acetate group, the statistical chain length is obtained. Some aliphatic OH groups may remain unmodified after oxyalkylation and would falsify the value of the statistical chain length since they are acetylated as well. It is possible to distinguish between unreacted aliphatic OH groups and grafted aliphatic OH groups in the ^31^P-NMR spectrum. For the calculation of the average chain length, the CH_3_ group of the PPG ether is related only to the PPG repetition unit. The different chain lengths of L-PO can be multiplied by the molecular weight of the PO repetition unit to calculate the molecular weight of the grafted chains (*M*_chain_). The mass of the grafted PPG chain *m*_chain_ is obtained by multiplication of *m*_chain_ by the amount of substance of PB1000 n_PB1000_. These values can be used for the determination of the theoretical yield (sum of *m*_chain_ and *m*_PB1000_) and the practical outcome of L-PO (based on chain length). Furthermore, a corrected OH number can be obtained as well (for results, see the Supporting Information). The grafting with PPG can be confirmed by GPC measurements as well. A growth of the molecular weight from 900 g·mol^–1^ for PB1000 to 2600–3400 g·mol^–1^ for L-PO samples is obtained. GPC data of all samples is given in Table S9.

**Table 1:** Results of Oxyalkylation Experiments

experiment	L-PO 1^a^	L-PO 2	L-PO 3	L-PO 4
yield m/m L-PO [wt %]^b^	32	40	45	52
yield m/m PO homopolymer [wt %]^b^	29	46	41	28
yield m/m insoluble part [wt %]^b^	39	14	14	20
OH number [mmol·g^–1^]	2.69	3.11	2.29	2.23
lignin content from. ^31^P-NMR [%]	41	41	30	29
statistical chain length [−]	5.84	3.77	5.77	8.62
average chain length [−]	6.79	4.86	7.53	10.86
yield L-PO [%]^c^	38	54	52	40
viscosity at 25 °C [Pa·s]	4.9 ± 0.1	6.7 ± 0.1	11.1 ± 0.4	3.7 ± 0.1

a For L-PO 1 a PB1000 sample with an OH number of 6.21 mmol·g^–1^ was used.

b Based on weight.

c Based on average chain length.

Although L-PO 1 has a high lignin content (41%), this PB1000 batch was less reactive during oxyalkylation because the mass fraction (yield by weight) of the insoluble part (39 wt %) was higher than the mass fraction of L-PO 1 (32 wt %). L-PO 2–4 show that longer PPG chains increase the mass fraction of L-PO from 40 to 52 wt % during oxyalkylation but decrease the lignin content from 41 to 29%. The insoluble residues of L-PO 2–4 syntheses remain nearly constant (14–20 wt %). Due to mixing dead zones in the autoclave, a part of PB1000 does not react and remains an insoluble residue. The reactivity of different lignin types during oxyalkylation is discussed contrarily in the literature.^12,13,19^ However, our oxyalkylation experiments can confirm that, on the one hand, with a lower PO/lignin ratio, a higher amount of insoluble residue is obtained.^19^ On the other hand, an increasing amount of insoluble residue decreases the homopolymer mass fraction.^12^ Based on liquid–liquid extraction with *n*-hexane, PO homopolymer contents are reported to be between 25 and 30 wt % for soda lignins^12^ and 44 and 48 wt % for sulfur-free lignins.^40^ The homopolymer content in our experiments varies between 28 and 45 wt %, corresponding to the values given in the literature. With viscosities below 20 Pa·s, the synthesized L-PO samples already fulfill the requirement for 3D-printing experiments on a hot lithography system.^39^

The oxyalkylation of PB1000 with PO leads to the following advantages: (i) improved solubility in organic solvents and resin formulations, (ii) decreased viscosity, and (iii) homogenization of different functional hydroxyl groups of the polymer. Due to their radical inhibition capacity, the transformation of phenolic OH groups is of particular importance.

After characterization, the propoxylated lignin is further modified with methacrylate groups according to the procedure presented by Hajirahimkhan et al.^17^ Here, methacryloyl chloride (MAC) is used as an esterification agent together with triethylamine (TEA) as an acid scavenger to convert the L-PO into a photopolymerizable macromonomer (Figure 1). The synthetic procedure is described in the Experimental Section, and the schematic of the procedure is given in Figure S5. The methacrylated propoxylated lignin (L-PO-MAC) is characterized by FTIR spectroscopy, NMR spectroscopy, and rheology. To evaluate the efficiency of L-PO-MAC samples in photopolymerization, real-time-NIR-photorheology experiments^38^ are performed before its application in 3D printing.

L-PO 1–4 were methacrylated to the corresponding L-PO-MAC 1–4 as described above, and the results are listed in Table 2. The theoretical yield of L-PO-MAC samples can be calculated with eq 1. Dividing the theoretical yield by the obtained weight of L-PO-MAC leads to the yield of the second synthetic step for a quantitative conversion of all OH groups (degree of substitution (DS) = 100%). Since a small amount of OH groups remain unreacted, the theoretical yield can be corrected by multiplication with the conversion obtained from quantitative ^31^P-NMR spectroscopy. The conversion of OH groups into reactive double bonds can be calculated with eq 2. The lignin content in L-PO-MAC is obtained by multiplication of the conversion term and the lignin content of L-PO.

**Table 2:** Results of Methacrylation of L-PO Samples

experiment	L-PO-MAC 1	L-PO-MAC 2	L-PO-MAC 3	L-PO-MAC 4
yield [%]	84	85	94	91
OH number [mmol·g^–1^]	0.16	0.21	0.19	0.24
conversion OH groups [%]	94	93	92	89
lignin content [%]	39	38	28	26
viscosity at 25 °C [Pa·s]	2.2 ± 0.1	2.5 ± 0.1	4.8 ± 0.0	1.7 ± 0.0
viscosity at 40 °C [Pa·s]	0.8 ± 0.0	0.9 ± 0.0	1.6 ± 0.0	0.6 ± 0.0

L-PO-MAC 1–4 products are obtained in high yields (84–94%), and the conversion of the OH groups into reactive double bonds is in the range between 89 and 94%. With methacrylation, further growth of the molecular weight is observed (Table S9). The *M*_n_ of methacrylated lignin samples was determined to be between 2700 and 3800 g·mol^–1^. The quantitative ^31^P-NMR spectrum (Figure 2a) shows a decrease of the different functional OH groups toward 0. Aliphatic OH groups, which are not grafted with PPG chains, are converted into methacrylate groups as well. The amounts of unreacted aliphatic OH groups in L-PO-MAC samples are mainly OH groups from grafted chains. With methacrylation, the lignin content in L-PO-MAC shows a decrease of 2–3% from 41–29 to 39–26%. In the ^1^H-NMR spectrum (see Figure S7), the appearance of two new signals is observed, which can be assigned to the olefinic protons from the methacrylate group. Structural changes can be monitored qualitatively in the FTIR spectrum (Figure 2b). Esterification is indicated by the disappearance of the vibrational stretching peak of OH groups at 3458 cm^–1^ and the formation of a C=O stretching vibration peak at 1717 cm^–1^. The viscosities of L-PO-MAC 1–4 could be further decreased (≤ 5 Pa·s), which is beneficial for the preparation of samples for photopolymerization and 3D printing. With low viscosities, the addition of solvents or resins as reactive diluents can be avoided, which is a big advantage over the current state-of-the-art applications. In the work of Hajirahimkhan et al., up to 31 wt % methacrylated lignin was mixed with 38 wt % methacrylic acid as a reactive diluent, tetra(ethylene glycol)diacrylate (TEGDA) as a cross-linker, and 5 wt % 2,2-Dimethoxy-2-phenylacetophenone (DMPA) as a photoinitiator (PI).^17^ These mixtures were used for UV coatings with a sample thickness of 15 μm. Sutton et al. successfully printed lignin-based formulations by SLA with a methacrylated lignin content of up to 15 wt %^33^ However, 2 resin bases (ethoxylated pentaerythritol tetraacrylate—SR494, aliphatic urethane acrylate—Ebecryl 8210) and a reactive diluent (monofunctional urethane acrylate—Genomer 1122) together with diphenyl(2,4,6-trimethylbenzoyl)phosphine oxide (PL-TPO) as PI had to be used. In our work, the two-step synthesis procedure leads to a liquefied lignin monomer with improved solubility in organic solvents and resin formulations. The lignin content in the final material remains high, and due to the low viscosity of our methacrylated lignin, only a photoinitiator has to be added for photopolymerization experiments.

L-PO-MAC 1–3 (18 wt % L-PO-MAC 1, 36 wt % L-PO-MAC 2, and 46 wt % L-PO-MAC 3) were unified into an L-PO-MAC master mixture and characterized as a new component by ^31^P-NMR spectroscopy and rheology. The L-PO-MAC master mixture has a viscosity of 3.9 ± 0.1 Pa·s at RT (1.4 ± 0.0 Pa·s at 40 °C), an OH number of 0.19 mmol·g^–1^, and a calculated lignin content of 33%. The remaining L-PO 4 batch shows the lowest OH conversion and lignin content and therefore was only used for one-layer photopolymerization experiments by real-time-NIR-photorheology.^38^

### Real-Time-NIR-Photorheology

**Figure 3:**
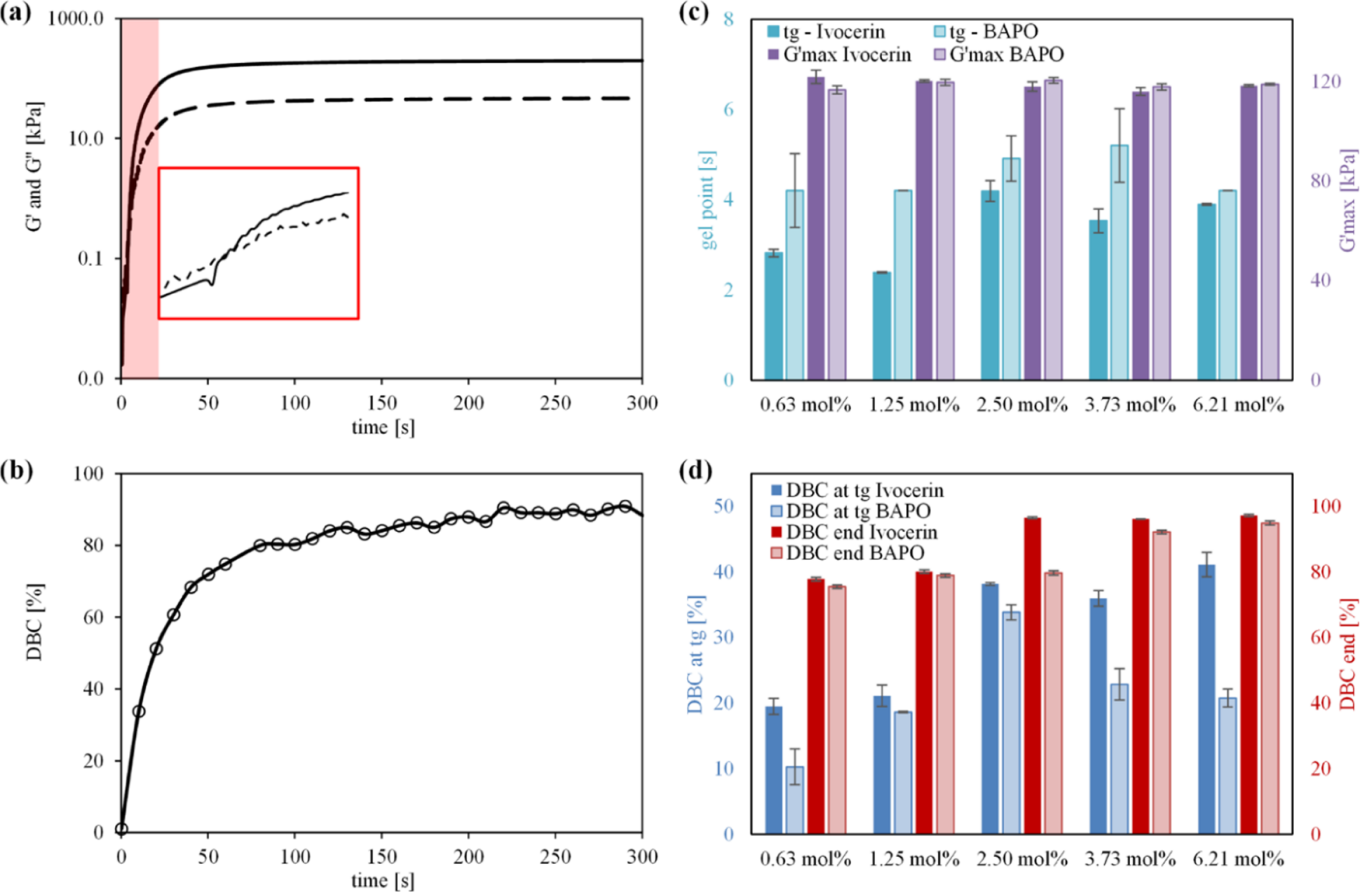
Real-time-NIR-photorheology experiments of L-PO-MAC formulations. Exemplary photorheology curves of the L-PO-MAC master mixture with 2 wt % Ivocerin as PI: (a) Storage modulus *G*′ (−) and loss modulus G″ (- -). (b) The double bond conversion DBC curve. Photorheology results of L-PO-MAC 4 formulations with different amounts of PI. (c) Gel point *t*_g_ and maximum storage modulus *G*′_max_. (d) Double bond conversion DBC at gel point *t*_g_ and final conversion DBC_end_ (bottom).

L-PO-MAC samples are characterized by real-time-NIR-photorheology.^38^ This method allows the in situ characterization of photopolymerizable resins concerning mechanical information about curing (e.g., gelation, increase in moduli, polymerization-induced shrinkage stress) and chemical conversion via IR spectroscopy. Due to the dark brown color of lignin samples, UV light is limited in penetration depth; therefore, the sample thickness had to be reduced (UV spectrum of L-PO-MAC master mixture, Figure S8) to 50 μm, which is a good trade-off between commonly used layer thicknesses during SLA but still enables the real-time monitoring of double bond conversion by IR spectroscopy. The sample preparation and measuring procedure are explained in detail in the Experimental Section. The evaluation of recorded storage modulus *G*′ and loss modulus *G*″ curves (Figure 3a) provide information about the gel point *t*_g_ and the maximum storage modulus *G*′_max_ of a formulation. The values for *t*_g_ are determined by the intersection of *G*′ and *G*″. The results for *G*′_max_ are obtained using the average of the last 15 points of *G*′. Double bond conversion of the samples is determined by recording a set of IR spectra and integrating the methacrylic peak (6080–6250 cm^–1^) over reaction time. The methacrylic peak ratio from the start to the end of a measurement gives the double bond conversion (DBC) (Figure 3b).

Bis(4-methoxybenzoyl)diethylgermanium (Ivocerin from Ivoclar Vivadent) and bis(2,4,6-trimethylbenzoyl)-phenylphosphine oxide (Irgacure 819, BAPO from Ciba Specialty Chemicals) are Norrish type I initiators that show a red-shifted (bathochromic) absorption behavior.^56^ They are used as PIs because they are suitable to initiate radical photopolymerization in colored formulations and filled systems. Preliminary studies of L-PO-MAC within our group showed good curing results with 2 wt % Ivocerin.

A first photorheology study was performed with L-PO-MAC 4 and different amounts (0.6–6.2 mol %) of either Ivocerin or BAPO as photoinitiators (Figure 3c,d). The OH number of L-PO-MAC is subtracted from OH_L-PO_, and the resulting term is defined as the amount of substance *n*_ene_ for calculations of necessary PI amounts. For better comparability, PI amounts were calculated in mol % (for L-PO-MAC 4: 2 wt % Ivocerin is equivalent to 2.5 mol %). The used amounts of materials and averaged *G*′ and DBC curves of all L-PO-MAC 4 formulations are given in Table S10 and Figure S9 in the Supporting Information.

The gel point is reached within 2–5 s of irradiation time. In comparison, L-PO-MAC formulations with Ivocerin as PI show faster gelation, a steeper increase of the curves, and higher DBC even at low PI concentrations. Although the storage modulus of the formulations varies before irradiation, the maximum storage modulus is in a similar region of 116–122 kPa after cross-linking, suggesting no major changes in the mechanical properties. The amount and type of PI seem to influence the rheological properties before cross-linking. As mentioned before, due to lignin’s dark color, suitable PIs and sometimes higher concentrations are necessary. The higher the amount of PI in a formulation, the more it is expected to directly influence the properties of the formulation and final material. The best results are obtained with Ivocerin as PI since Ivocerin absorbs light almost in the whole UV–vis light range (340–470 nm) of the broadband Hg lamp (320–500 nm). An increase of Ivocerin concentration up to 2.5 mol % has a positive effect on the DBC of L-PO-MAC 4. With 2.5 mol % for both PIs, the highest DBC values at the gel point were achieved, but the largest difference in the final DBC and delayed gelation was observed. A further increase to 6.2 mol % of Ivocerin (here equivalent to 5 wt %) leads to comparable results regarding DBC. Using BAPO as a photoinitiator for photopolymerization of L-PO-MAC, at least 3.7 mol % is necessary to achieve a final double bond conversion over 90%. The data of all L-PO-MAC 4 formulations is available in the Supporting Information (Table S11). L-PO-MAC 1–3 formulations and L-PO-MAC master mixture were characterized by photorheology as well (Figures S10 and S11 and Table S12). Two weight percent Ivocerin was used as PI for each formulation since it showed the best results. *G*′_max_ values between 194 and 209 kPa and final DBC over 87% are obtained. Although the L-PO-MAC master mixture shows the lowest DBC at *t*_g_, the resulting final DBC is still 90%.

In the work of Sutton and co-workers, PL-TPO (0.4 wt %) was used as PI for SLA printing of formulations containing 0–15 wt % methacrylated lignin.^33^ The amount of PL-TPO was increased to 0.84 wt % and higher in later studies for UV curing of formulations containing 5 wt % methacrylated lignin.^34^ In this study, no information about the DBC values is mentioned for these rather low PI amounts. Hajirahimkhan et al. used 5 wt % DMPA for their photopolymerization experiments and determined the cure percentage by FTIR measurements. With an increasing amount of methacrylated lignin, the cure percentage increases. The formulation containing 31 wt % methacrylated lignin shows a DBC of 68%.^18^ Bassett and co-workers used the lignin-derivative vanillin to synthesize a bio-based resin (vanillin content 34.8%) for SLA printing. The resin contained vanillin methacrylate (MV) and glycerol dimethacrylate (GDM) (molar ratio 1:1) and 2 wt % diphenyl(2,4,6-trimethylbenzoyl)phosphine oxide (TPO) as the photoinitiator. It was possible to print specimens with 100 μm layer thickness with a conversion of 66%. The post-curing of the yellow specimens could increase the cure percentage to 89%.^57^ Compared to literature-known formulations, the amount of photoinitiator in our photorheology experiments is mainly the same range, but a higher double bond conversion of methacrylated lignin is achieved. The photorheology experiments of L-PO-MAC 4 proved similar reactivity using Ivocerin or BAPO as PI but slightly higher DBC for Ivocerin. The obtained results indicate an amount of at least 2 wt % Ivocerin or 3 wt % BAPO for 3D-printing experiments. Due to the rather high price of Ivocerin, BAPO is a suitable alternative since formulation amounts of around 80 g are required for this study’s 3D-printing experiments. Usually, 3D-printed specimens are post-cured thermally and/or by UV light to ensure the reaction of all reactive groups. For lignin-based photopolymers, post-curing is rather difficult due to the dark brown color. Here, it must be realized while printing. If a 3D-printed layer is thin enough, UV light can penetrate the surface and post-cure the layer below. Since SLA printer light sources work on a specific wavelength (e.g., 405 or 460 nm), the efficiency of photopolymerization of a system can differ. The efficiency of a photoinitiator strongly depends on the employed system and parameters like absorption behavior, radical cleavage, and the reactivity of the formed radicals.^58^ The concentration of a photoinitiator and the sample thickness of the cured formulation are important as well.^59^ Regarding 3D printing, a lower photoinitiator efficiency at a certain wavelength would lead to longer UV light exposure times to increase the curing time.

### 3D Printing of Modified Lignin

For successful processing by L-AMTs, photopolymer resins have to provide certain characteristics. The resin must be curable by the printer’s wavelength, which can be achieved by adding an appropriate photoinitiator. An important step for introducing new materials in 3D printers is testing and adjusting their cure depth to obtain precise structures by well-defined layers without over-curing. For dark brown lignin resins, a cure depth of about 2–3 times the final layer height is desired to ensure the polymerization of the cured layers during the printing process and the formation of homogeneous printed parts without anisotropy. Another advantage is the post-curing of a printed layer while curing the next layer. Since the post-curing of lignin resins is difficult due to their dark brown color and the limited penetration depth of the UV light, it is mandatory that SLA 3D-printed parts already reach a high chemical conversion and their final mechanical and thermal properties during the printing process.

The commercial laser printer Caligma 200 of Cubicure^39^ was used for 3D-printing experiments of the L-PO-MAC master mixture. The significantly higher energy input using a laser as a light source was considered to be beneficial for curing the neat L-PO-MAC mixture. However, the penetration depth of light and kinetics of the initiated polymerization reaction greatly differ using a laser light source compared with a mercury broadband lamp. First exposure tests on the laser printer Caligma 200 with a wavelength of 405 nm showed the formation of a very thin film of a neat L-PO-MAC mixture with 3 wt % BAPO as the photoinitiator (Figure S12, top). The subsequent modification of printing parameters and alteration trials of the used photoinitiator (BAPO, Ivocerin) resulted in the formation of detachable and measurable, well-resolved platelets with a thickness of 60–90 μm (Figure S12, bottom). With the achieved 60–90 μm layer thickness during the exposure tests, a final layer height of 25 μm for 3D-printing trials was chosen. To achieve a bio-based, sustainable formulation, the L-PO-MAC content (which contains 33% of renewable lignin) was kept as high as possible, and the formulation was kept as neat as possible (only L-PO-MAC master mixture + 3 wt % BAPO). No petrochemical co-monomers or process additives were added.

**Figure 4:**
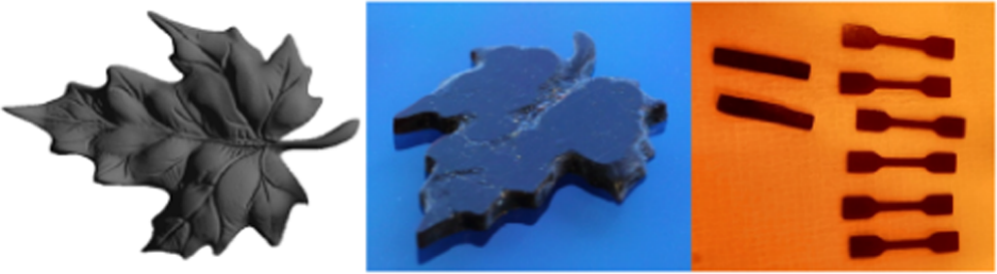
3D-printing experiments: CAD render (left), printed show part (middle), and test specimen (right). Note: photos of the 3D-printed parts were made under different light conditions (white and orange light laboratory environment). The printed maple leaf has the same dark brown color as the test specimen. The STL file is available free of charge on the Thingiverse website.^60^

Subsequent print jobs were conducted, which resulted in 3D-printed test specimens for tensile and DMTA measurements and a maple leaf (Figure 4). For the 3D printing of more complex parts with more layers, process parameters need to be further optimized, and the addition of process additives (e.g., adhesion promotor) might be necessary.

### Characterization of Printed Lignin Specimen

**Figure 5:**
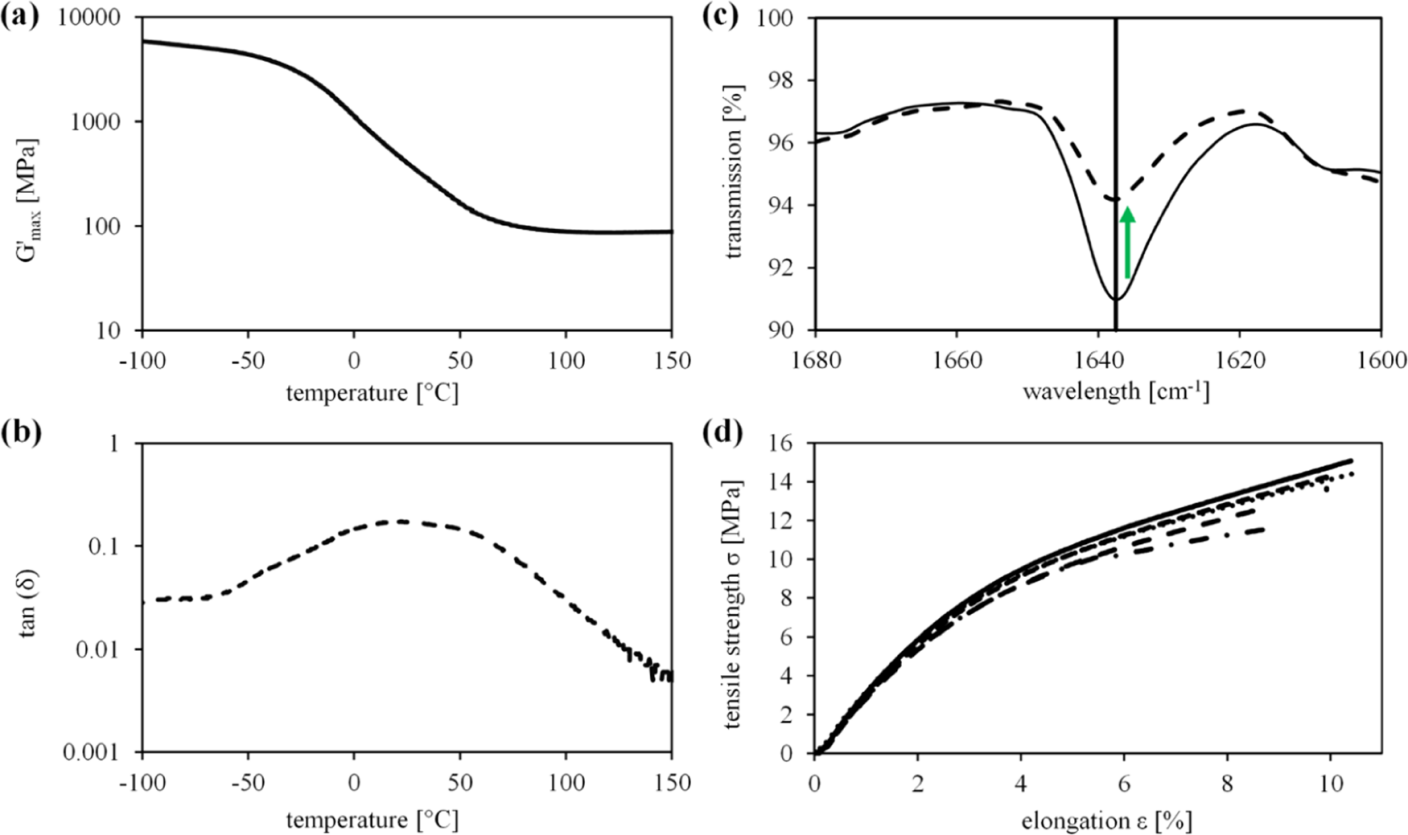
DMTA results of the printed L-PO-MAC specimen: (a) *G*′_max_ and (b) tan δ curves. (c) The ATR-IR signal of available double bonds before (−) and after curing (−). (d) Tensile test results of the printed L-PO-MAC specimen.

Dynamic mechanical thermal analysis (DMTA) was used to determine the glass transition temperature *T*_g_ of the L-PO-MAC photopolymer printed by hot lithography. The corresponding curves of *G*′ and tan δ are shown in Figure 5. The *T*_g_ is determined from the maximum of tan δ curve, which is at 21.95 °C for the L-PO-MAC mixture with a storage modulus of 448.95 MPa at *T*_g_. The formulation was characterized by ATR-IR measurement before and after printing. A decrease of the ν C=C signal at 1638 cm^–1^ indicates that ∼50% of the available double bonds were converted during the printing process (Figure 5). This result deviates from photorheology experiments shown in Figure 3. To achieve better comparability and more accurate results, transmittance measurements might be beneficial, but reduced signal strength due to the dark color of the lignin needs to be considered. The tensile test results give Young’s modulus of *E* = 220 MPa, a tensile strength of σ = 13.5 MPa, and elongation at break of ε = 9.7% for the as-printed L-PO-MAC with a ∼10% deviation of the results. Post-processing trials via UV-light curing (*E* = 212 MPa, σ = 14 MPa, ε = 8.6%), as well as thermal treatment for 4 h at 150 °C, did not result in divergent properties indicating a higher conversion than evaluated via ATR-IR. An extensive post-processing study would be required to confirm results from conversion evaluation and reach a fully cured part.

**Figure 6:**
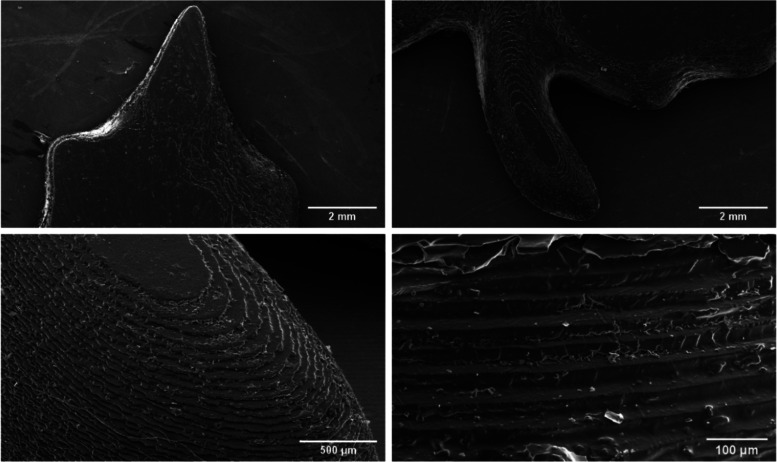
SEM pictures of 3D-printed show parts.

Scanning electron microscopy (SEM) pictures (Figure 6) of the printed show part, on the one hand, shows a smooth surface but, on the other hand, mostly rather rough layer edges due to the brittleness of the material. For further investigations, the focus should be put on a bespoke post-processing procedure to ensure optimized end-part properties. After SEM measurements, the 3D printed show part was analyzed with a digital microscope to get information about the height profile of the specimen, enabling layer counting. The analyzed area and its height profile are shown in Figure S13 in the Supporting Information.

## Conclusions

In this work, we synthesized a photopolymerizable macromonomer based on wheat straw soda lignin in a two-step procedure. After advanced characterization with qualitative and quantitative methods, the methacrylated lignin was 3D printed via hot lithography, a 3D-printing technology is well-suited for photopolymerizable resins with viscosities > 2 Pa·s. For the first time, a light-curable lignin derivative with a lignin content >30% was successfully 3D printed via vat photopolymerization without any reactive diluents. Overall, a lignin content of greater than 30% translates to a great improvement over state-of-the-art solutions known in the literature. This outstanding result is a motivating proof of concept and shows the high potential for the employment of lignin as an abundant biopolymer for lithography-based 3D printing. Nevertheless, the UV absorption of the dark brown methacrylated lignin remains the critical limitation, but potential bleaching of lignin as a solution could maybe yield higher contents of lignin in 3D-printable resins in the future. Certainly, it is also a promising starting point for an in-depth evaluation of bio-based precursors as an alternative to nonrenewable derivatives for 3D printing. When now aiming for various applications in industry like consumer products (e.g., eyeglass frames), electronic parts, or art and design (e.g., decoration), the material performance (e.g., mechanical properties, low toxicity) needs to be adapted to meet the individual needs, but imperatively a high content of renewable material needs to be maintained.

## Supporting Information Available

^13^C-, 2D-HSQC-, and ^31^P-NMR spectra of PB1000; calculation of OH number and lignin content via ^31^P-NMR spectroscopy; determination of grafted chain length by ^1^H-NMR spectroscopy; schematic reaction scheme; calculations of theoretical yield and practical outcome of L-PO and L-PO-MAC; ^1^H-NMR spectra of L-PO and L-PO-MAC; UV–Vis spectrum of L-PO-MAC, GPC data, real-time-NIR-photorheology data of L-PO-MAC formulations; and 3D printing and characterization of the printed specimen (PDF)
