# Supplementary material for: Synthesis of a Liquid Lignin-Based Methacrylate Resin and Its Application in 3D Printing without Any Reactive Diluents
Source: Biomacromolecules. 2023 Mar 17;24(4):1751–62. doi: 10.1021/acs.biomac.2c01505 (PMC10091419; doi:10.1021/acs.biomac.2c01505)
Supplement: Supplementary file 1 — bm2c01505_si_001.pdf [file bm2c01505_si_001.pdf]

Supporting Information

Synthesis of a liquid lignin-based methacrylate resin and its application in 3D printing without any reactive diluents

Sarah Keck[1], Olga Liske[1], Konstanze Seidler[2], Bernhard Steyrer[2], Christian Gorsche[2], Simone Knaus[1], Stefan Baudis[1*]*

[1] Institute of Applied Synthetic Chemistry, Technische Universität Wien, Getreidemarkt 9/163, 1060 Vienna, Austria

[2] Cubicure GmbH, Gutheil-Schoder-Gasse 17, Tech Park Vienna, 1230 Vienna, Austria

** corresponding authors e-mail: stefan.baudis@tuwien.ac.at; konstanze.seidler@cubicure.com*

CONTENTS

Characterization of PB1000 by ^{13}C and 2D HSQC NMR spectroscopy I
Calculation of OH number via quantitative ^{31}P-NMR-spectroscopy V
Calculation of lignin content via quantitative ^{31}P-NMR-spectroscopy VII
Schematic reaction scheme VIII
Determination of grafted chain length by ^{1}H-NMR-spectroscopy IX
Calculation of theoretical yield and practical outcome of L-PO XI
Calculation of theoretical yield and practical outcome of L-PO-MAC (DS=100%) XII
^{1}H-NMR-spectrum and assignment of methacrylated lignin L-PO-MAC XIII
UV-Vis spectrum of L-PO-MAC XIII
Gel permeation chromatography (GPC) data XIII
Real time-NIR-photorheology data of L-PO-MAC formulations XIV
3D printing and characterization of the printed specimen XVII
References XVIII

Characterization of PB1000 by ^{13}C and 2D HSQC NMR spectroscopy

^{13}C- and 2D-HSQC-NMR-spectra were recorded according to the procedure described in the main manuscript. *Mestrenova Version 12.0.0-20080* was used for the analysis of the NMR spectra. First, a manual phase and baseline correction (polynomial fit) was performed for each spectrum. Afterward, the signal of DMSO-d_6 was referenced to 2.50 ppm (^{1}H-NMR) and/or 39.52 ppm (^{13}C-NMR). The signals were assigned according to literature. [1–5] The 2D-HSQC-NMR results are given in **Figure S1** and **Table S1**. ^{13}C-NMR results are shown in **Figure S2** and **Table S2**.

Table S1: Assignment of the ^{13}C-^{1}H cross-signals in the 2D HSQC spectrum of PB1000. [1,3,5]

label	δ_C/ δ_H (ppm)	assignment [(a)]
PCA_α /FA_α	144.10/7.52	C_α-H_α in *p*-coumarate (PCA) and ferulate (FA)
$PCA_{2,6}$	129.89/7.52	$C_{2,6}$-$H_{2,6}$ in *p*-coumarate (PCA)
$H_{2,6}$	127.76/7.20	$C_{2,6}$-$H_{2,6}$ in *p*-hydroxyphenyl units (H)
G_6	119.07/6.74	C_6-H_6 in guaiacyl units (G)
G_5	114.90/6.70	C_5-H_5 in guaiacyl units (G)
G_2	111.74/6.90	C_2-H_2 in guaiacyl units (G)
FA_2	110.70/7.39	C_2-H_2 in ferulate (FA)
$S'_{2,6}$	106.29/7.24	$C_{2,6}$-$H_{2,6}$ in oxidized (C_α=O) syringyl units (S’)
$S_{2,6}$	104.87/6.60	$C_{2,6}$-$H_{2,6}$ in syringyl units (S)
X_1	101.72/4.28	C_1-H_1 in *β*-D-xylopyranoside
$A_{\beta(S)}$	86.21/4.12	C_β -H_β in *β*-*O*-4’ substructures linked to S units (A)
B_α	85.03/4.63	C_α-H_α in resinol substructures (B)
$A_{\beta(G)}$	84.33/4.30	C_β -H_β in *β*-*O*-4’ substructures linked to a G unit (A)
$A_{\beta(H)}$	83.80/4.44	C_β -H_β in *β*-*O*-4’ substructures linked to a H unit (A)
BE_α	81.37/4.76	C_α-H_α in benzyl ether LCC structures
X_4	75.21/3.53	C_4-H_4 in *β*-D-xylopyranoside
X_3	73.72/3.27	C_3-H_3 in *β*-D-xylopyranoside
$X2_2$	73.97/4.43	C_2-H_2 in 2-*O*-acetyl-*β*-D-xylopyranoside
X_2	72.61/3.07	C_2-H_2 in *β*-D-xylopyranoside
$A_{\alpha(s)}$	71.64/4.90	C_α-H_α in *β*-*O*-4’ substructures linked to a S-unit (A)
$A_{\alpha(G)}$	71.38/4.80	C_α-H_α in *β*-*O*-4’ substructures linked to a G-unit (A)
B_γ	70.73/4.16	C_γ- H_γ in resinol substructures (B)
	70.28/3.84	
X_5	63.39/3.90	C_5-H_5 in *β*-D-xylopyranoside
	62.82/3.20	
A_γ	59.75/3.38	C_γ- H_γ in *β*-*O*-4’ substructures (A)
	59.54/3.64	
-OCH_3	55.69/3.75	C-H in methoxyls
B_β	53.65/3.07	C_β -H_β in resinol substructures (B)

[(a)] assignment abbreviations: G, guaiacyl unit; S, syringyl unit; H, *p*-hydroxyphenyl unit; PCA, *p*-coumarate; FA, ferulic acid/ferulate; BE, benzyl ether; X, *β*-D-xylopyranoside.

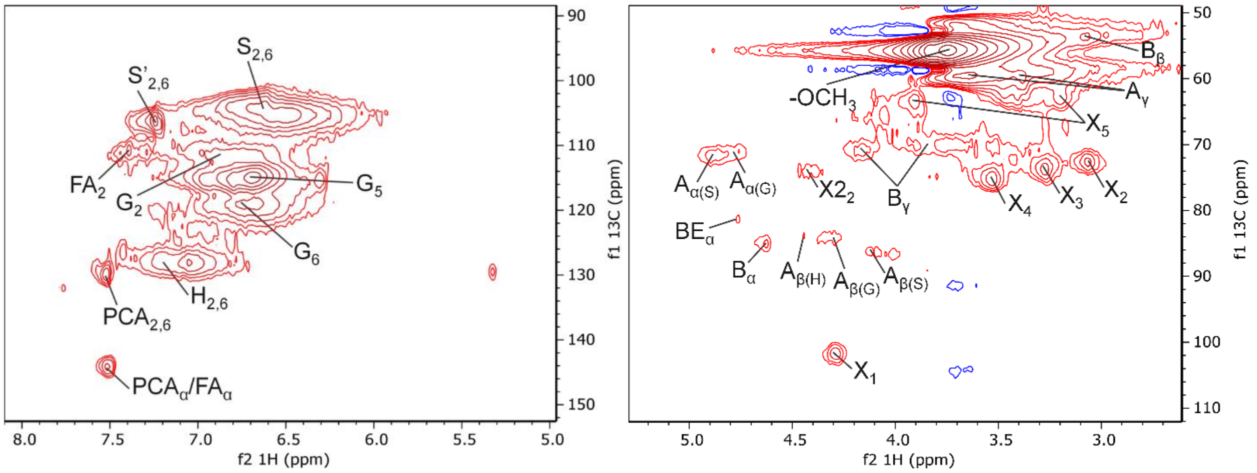

Figure S1: Aromatic (left) and side-chain (right) regions in the 2D-HSCQ-NMR spectrum of PB1000.

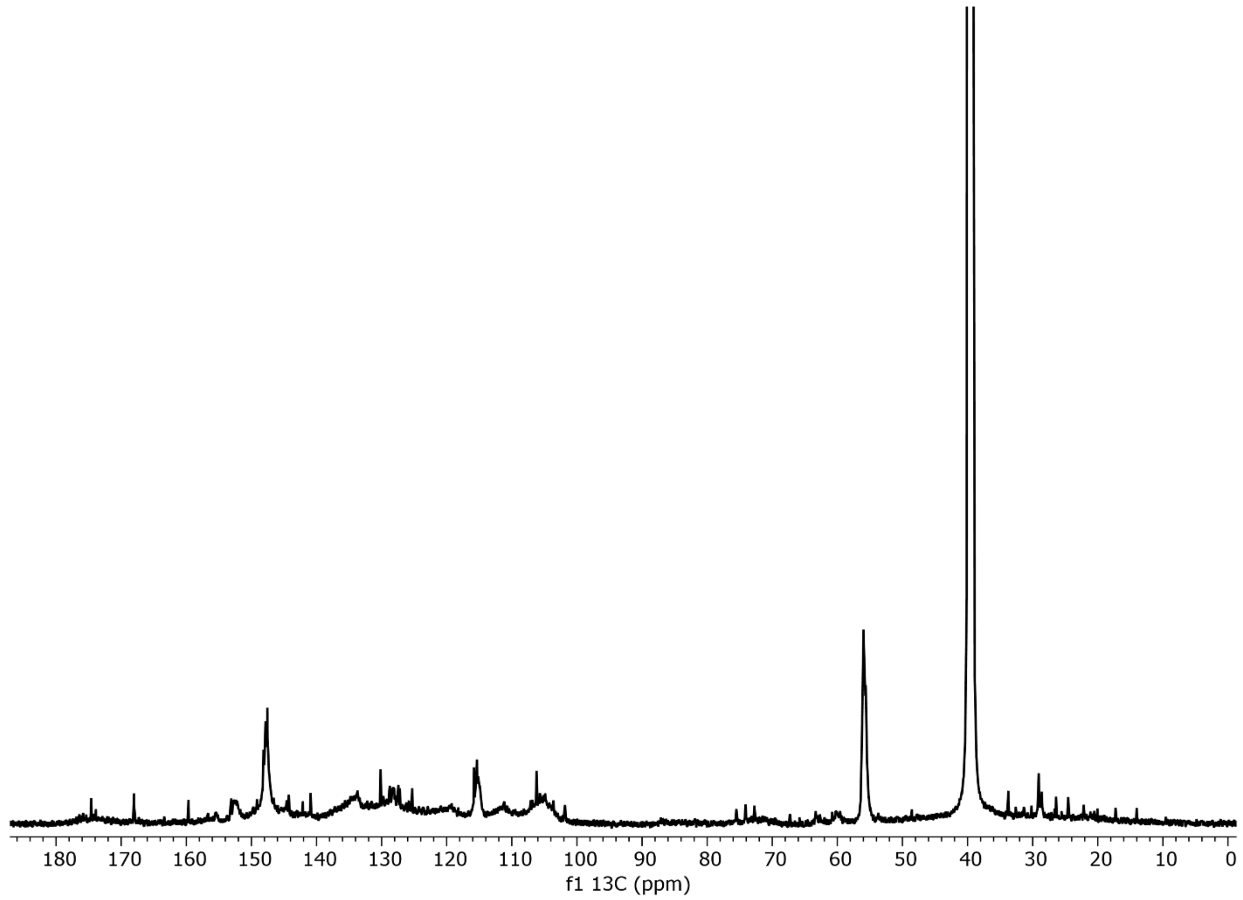

Figure S2: ^{13}C-NMR spectrum of PB1000 in DMSO-d_6.

Table S2: Chemical shift value (δ,ppm) and signal assignment of ^{13}C-NMR spectrum of PB1000. [2,4]

ppm	assignment [(a)]	ppm	assignment
174.60	-COOH in aliphatic acids or esters	111.15	C2 in G
168.04	C_γ in FA ether, C-γ in PCA ester	110.70	C_2 in FA
159.67	C_4 in PCA ester	106.87	$C_{2,6}$ in S with α-CO
152.36	$C_{3,5}$ in S etherified	106.20	$C_{2,6}$ in S with α-CO [(b)]
149.16	C_4 in G etherified	105.71	$C_{2,6}$ in S with α-CO [(b)]
148.23	C_3 in G	103.62	$C_{2,6}$ in S
148.15	C_3 in G	101.84	C_1 in β-D-xylopyranoside [(b)]
147.95	C_3 in G	75.51	C_4 in β-D-xylopyranoside
147.83	C_3 in G	74.11	C_3 in β-D-xylopyranoside
147.69	C_3 in G	72.73	C_2 in β-D-xylopyranoside
147.55	C_3 in G	63.33	C_5 in β-D-xylopyranoside
144.59	C_α in PCA ester	60.28	C_γ in β-O-4'; β-aryl ether
144.26	C_α in FA ether	59.56	C_γ in β-O-4'; β-aryl ether
133.71	C_1 in S and G non-etherified	55.98	OCH_3
130.17	$C_{2,6}$ in PCA ester	53.73	C_β in resinol structures [(b)]
129.73	$C_{2,6}$ in PCA ester	33.72	CH_3 in ketones (conj.) or in aliphatic
128.75	$C_{2,6}$ in H [(b)]	32.61	CH_3 in ketones (conj.) or in aliphatic
128.07	$C_{2,6}$ in H	31.35	CH_3 in ketones (conj.) or in aliphatic
127.94	$C_{2,6}$ in H	30.17	CH_3 in ketones (conj.) or in aliphatic
127.42	$C_{2,6}$ in H	29.06	CH_2 in aliphatic side chain
127.20	$C_{2,6}$ in H	28.81	CH_2 in aliphatic side chain
125.81	C_1 in PCA ester	28.61	CH_2 in aliphatic side chain
125.31	C_1 in PCA ester	27.12	CH_2 in aliphatic side chain
122.91	C_6 in FA ester	26.63	CH_3 or CH_2 in saturated side chains
119.24	C_6 in G	25.57	CH_3 or CH_2 in saturated side chains
118.22	C_6 in G	24.56	CH_3 or CH_2 in saturated side chains
115.81	$C_{3,5}$ in PCA	22.17	CH_3 or CH_2 in saturated aliphatic chain
115.36	$C_{3,5}$ in PCA	14.03	γ-CH_3 in n-propyl side chain
114.80	C_5 in G		

[(a)] assignment abbreviations: G, guaiacyl unit; S, syringyl unit; H, *p*-hydroxyphenyl unit; PCA, *p*-coumarate; FA, ferulic acid/ferulate [(b)] assignment by HSQC

Calculation of OH number via quantitative ^{31}P-NMR-spectroscopy

Lignin contains different functional hydroxyl groups such as aliphatic OH, phenolic OH, and carboxylic OH. The total OH number can be determined by titration or quantitative ^{31}P-NMR-spectroscopy. The advantage of ^{31}P-NMR-spectroscopy is the small sample amount that is necessary for measurement. Lignin samples are derivatized with the phosphorous agent 2-chloro-4,4,5,5-tetramethyl-1,3,2-dioxaphospholane (TMDP). Furthermore, cyclohexanol as internal standard (IS) and the relaxation agent chromium(III)acetylacetonate ($Cr(acac)_3$) are being used for ^{31}P-NMR-measurements.

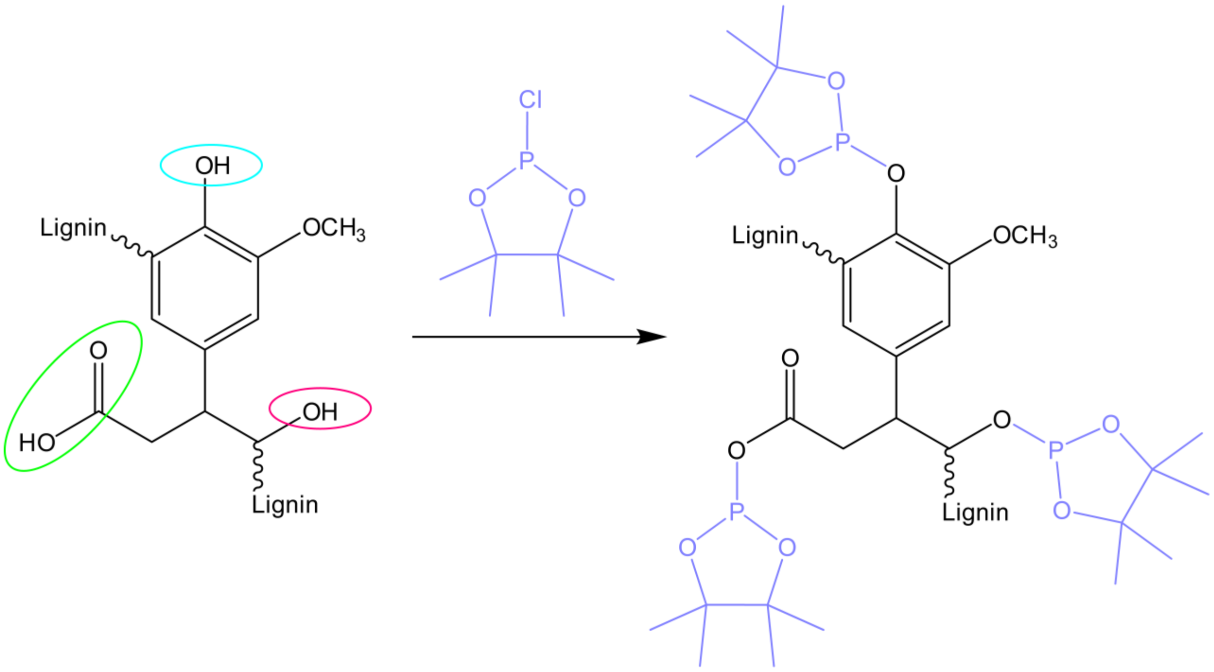

Figure S3: Phosphorylation of OH groups with TMDP agent for quantification of OH groups in lignin samples by ^{31}P-NMR-spectroscopy.

~30 mg lignin sample dissolved in 200 µL of a dimethylformamide (DMF)/pyridine mixture (1:1, v/v) in a glass vial and shaken with a *VELP Scientifica ZX4 Vortex mixer* (2 min, 2000 rpm). Since TMDP is moisture sensitive, the used solvents should be anhydrous. Then, 50 or 100 µL of a prepared cyclohexanol/$Cr(acac)_3$ mixture in pyridine (concentration: 40.58 mg·mL^{-1} cyclohexanol and 5.001 mg·mL^{-1} $Cr(acac)_3$) was added to the lignin sample and homogenized with the *Vortex mixer* (2 min, 2000 rpm). Separately, 400 µL deuterated chloroform ($CDCl_3$) and 50 or 100 µL TMDP were mixed and applied to the lignin sample solution. After a short mixture at room temperature (RT) the ^{31}P-NMR-spectrum was measured immediately. ^{31}P-NMR-spectra were recorded on a *Bruker Avance 400* NMR spectrometer with an *igated* mode (256 scans, 25 s relaxation time, duration 1 h 50 min). Using a 600 MHz spectrometer measuring time can be reduced to 128 scans. The used integration areas for lignin samples are listed in **Table S3**. An exemplary quantitative ^{31}P-NMR-spectrum of PB1000 with detailed information of the functional OH groups is shown in **Figure S4**.

Table S3: Functional group assignments for ^{31}P-NMR-spectra.[6,7]

**Functional group assignment$^{(a)}$**	**Integration area δ [ppm]**
TMDP	176.0
aliphatic OH	145.2 – 150.0
cyclohexanol (IS)	144.7 – 145.2
total phenolic OH	136.5 – 144.5
condensed phenolic units	
G_{cond}	144.5 – 143.3
G_{cond}	142.0 – 141.2
uncondensed phenolic units	
S-OH	143.3 – 142.0
G-OH	140.5 – 138.6
H-OH	138.5 – 137.3
carboxylic OH	133.5 – 136.5
hydrolysis product of TMDP	132.2 and 15.9

$^{(a)}$ abbreviations: G, guaiacyl; G_{cond}, 5-condensed G units; H, *p*-hydroxyphenyl; S, syringyl

All ^{31}P-NMR-spectra were analyzed with *Mestrenova Version 12.0.0-20080* in the following way. First, a manual phase and baseline correction (polynomial fit) was performed. Afterward, all signals were referenced to the signal at 132.2 ppm and then the integral of the internal standard (cyclohexanol) at 145 ppm was set to 1. All other signals were integrated as described in **Table S3**. The following equation was used to calculate the OH number from integrals of specific regions in the ^{31}P-NMR spectrum. The total OH number is obtained by the sum of all OH numbers. Results of PB1000, L-PO, and L-PO-MAC samples are given in **Table S4**.

$$\mathrm{OH}\ [\mathrm{mmol}\cdot\mathrm{g}^{-1}] = \frac{\mathrm{c_{IS}}[\mathrm{mg}\cdot\mathrm{mL}^{-1}]\cdot\mathrm{V_{IS}}[\mathrm{mL}]\cdot\mathrm{I_{funct.group}}}{\mathrm{M_{IS}}\ [\mathrm{g}\cdot\mathrm{mol}^{-1}]\cdot\mathrm{I_{IS}}\ \cdot\mathrm{m_{sample}}\ [\mathrm{mg}]}\cdot 1000$$

c_{IS}: concentration of cyclohexanol standard [40.58 mg·mL^{-1}]

V_{IS}: volume addition of cyclohexanol standard [0.5 or 0.1 mL]

$I_{funct.group}$: integral of corresponding functional group in the defined integration area

M_{IS}: molecular weight of cyclohexanol [100.158 g·mol^{-1}]

I_{IS}: integral of cyclohexanol standard [integral = 1]

m_{sample}: weight of lignin sample [≈ 30 mg]

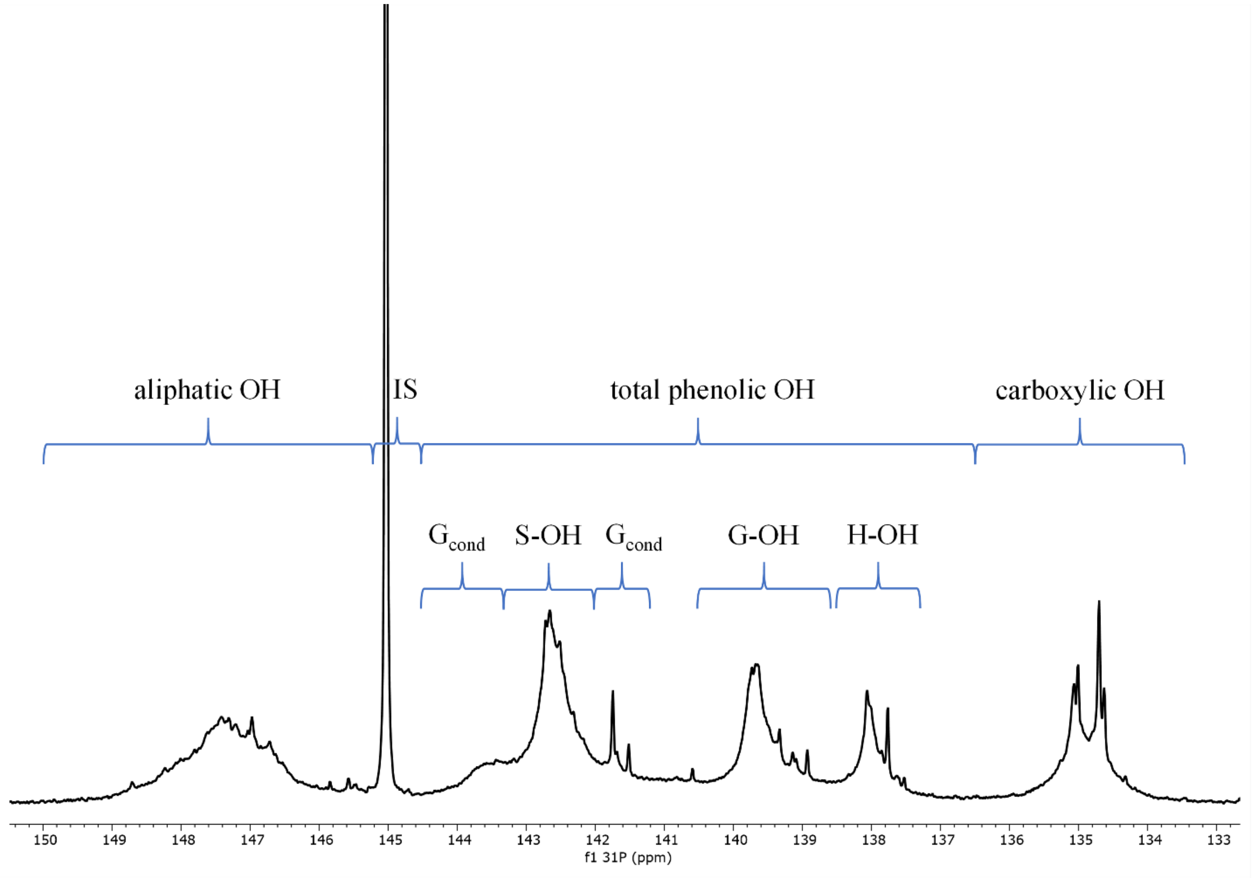

Figure S4: Quantitative ^{31}P-NMR-spectrum of PB1000 with assignment of the different functional OH groups.

Table S4: OH numbers of used lignin samples determined by quantitative ^{31}P-NMR-spectroscopy.

**PB1000*** 6.57 mmol·g^{-1}		**PB1000** 7.58 mmol·g^{-1}	
**L-PO 1*** 2.69 mmol·g^{-1}	**L-PO 2** 3.10 mmol·g^{-1}	**L-PO 3** 2.29 mmol·g^{-1}	**L-PO 4** 2.23 mmol·g^{-1}
**L-PO-MAC 1** 0.16 mmol·g^{-1}	**L-PO-MAC 2** 0.21 mmol·g^{-1}	**L-PO-MAC 3** 0.19 mmol·g^{-1}	**L-PO-MAC 4** 0.24 mmol·g^{-1}

Calculation of lignin content via quantitative ^{31}P-NMR-spectroscopy

The lignin content in L-PO and L-PO-MAC samples is determined by the OH number obtained from ^{31}P-NMR-spectroscopy. The results are given in **Table S5.**

$$\text{lignin content in L-PO [\%]} = \left(\frac{OH_{L-PO}}{OH_{PB1000}}\right) \cdot 100$$

$$\text{lignin content in L-PO-MAC [\%]} = \left(\frac{OH_{L-PO} - OH_{L-PO-MAC}}{OH_{PB1000}}\right) \cdot 100$$

OH_{PB1000} = OH number [mmol·g^{-1}] of unmodified wheat straw soda lignin ProtoBind1000

$OH_{L\text{-}PO}$ = OH number [mmol·g^{-1}] of propoxylated lignin L-PO

$OH_{L\text{-}PO\text{-}MAC}$ = OH number [mmol·g^{-1}] of methacrylated propoxylated lignin L-PO-MAC

Table S5: Lignin content in L-PO and L-PO-MAC samples calculated by OH number obtained from ^{31}P-NMR-spectroscopy.

L-PO 1*	L-PO 2	L-PO 3	L-PO 4
41 %	41 %	30 %	29 %
**L-PO-MAC 1**	**L-PO-MAC 2**	**L-PO-MAC 3**	**L-PO-MAC 4**
39 %	38 %	28 %	26 %

Schematic reaction scheme

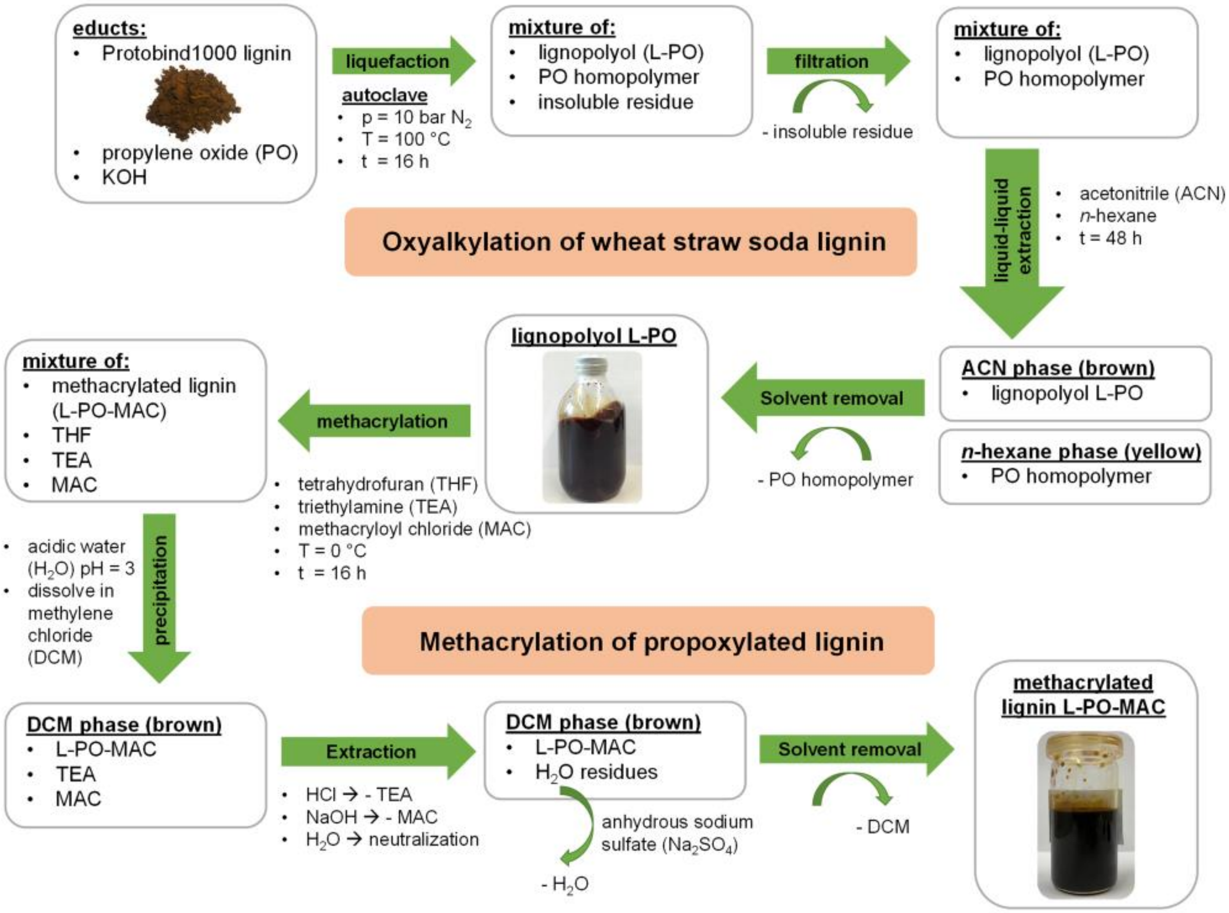

Figure S5: Scheme of synthetic pathway. First, PB1000 is oxyalkylated with propylene oxide under alkaline conditions to obtain a lignopolyol (L-PO). In the second step, lignopolyol is methacrylated with methacryloyl chloride (MAC) and triethylamine (TEA – catalyst and acid scavenger) into a photopolymerizable macromonomer L-PO-MAC.

Determination of grafted chain length by ^{1}H-NMR-spectroscopy

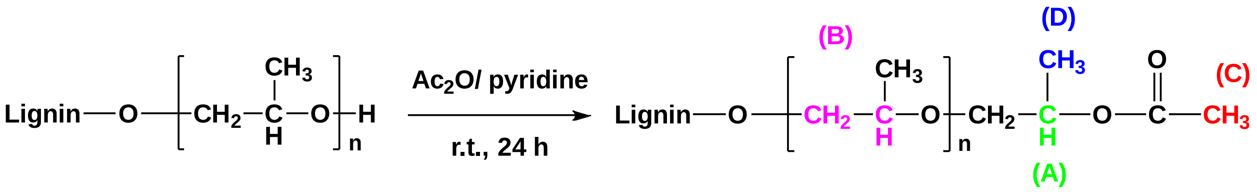

For determination of the grafted chain length, 1 g L-PO is acetylated with 8.1 mL pyridine (0.1 mol, 1 eq) and 5 mL acetic anhydride (0.1 mol, 1.6 eq). The mixture is stirred for 24 h at room temperature. After 24 h the acetylated L-PO was cooled with an ice bath and 50 mL ethanol was added in order to esterify the remaining excess of acetic anhydride. The solution was stirred for 30 min and the solvent was evaporated to dryness with a membrane pump vacuum (100 mbar) afterward. The procedure was repeated 3 times. The residue was extracted with a dichloromethane/water mixture and the organic phase was dried with sodium sulfate. After filtration, the product was dried with membrane pump vacuum. ^{1}H-NMR spectra were measured in 0.6 µL $CDCl_3$ with a *Bruker Avance 400* or *Bruker Avance 600* MHz spectrometer (16 scans). The ^{1}H-NMR spectrum of L-PO 1 is shown in **Figure S6**.

All ^{1}H-NMR-spectra of acetylated L-PO samples were analyzed with *Mestrenova Version 12.0.0-20080* in the following way. If necessary, a manual phase and baseline correction (polynomial fit) was performed. Afterward, the signal of $CDCl_3$ was referenced to 7.26 ppm and then the integral of the CH group from the chain end at 5 ppm was set to 1. All other signals were integrated as described in **Table S6**.

Table S6: Assignment for acetylated L-PO ^{1}H-NMR-spectra.

**assignment**	**integration area δ [ppm]**
-CH group at chain end	4.9 – 5.1
-$CHCH_2$- group of PPG ether backbone	3.1 – 4.0
-CH_3 of acetate group	1.8 – 2.1
-CH_3 group of PPG ether backbone	0.8 – 1.4

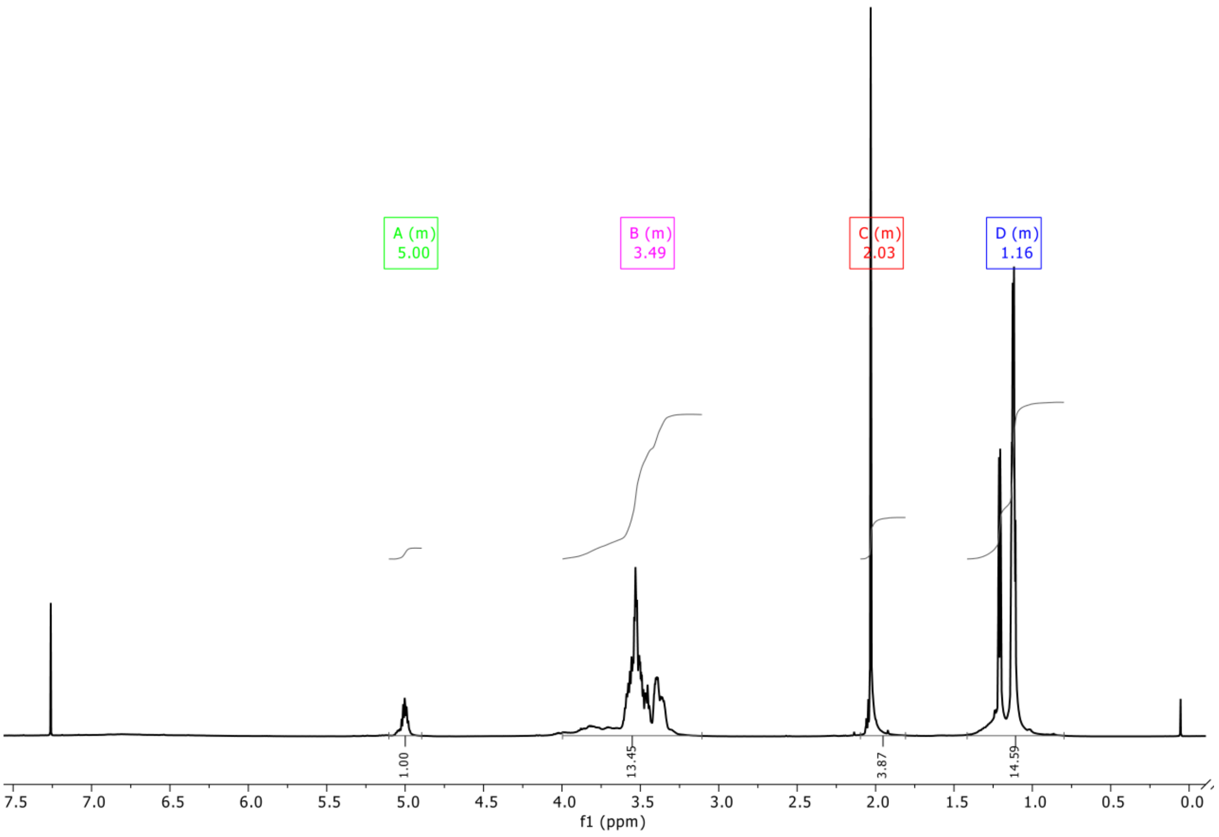

Figure S6: ^{1}H-NMR-spectrum of acetylated L-PO 1 measured in $CDCl_3$.

The statistical chain length $n_{stat.}$ of grafted PPG chains can be calculated by dividing integral (D) by integral (C). During oxyalkylation of PB1000 phenolic and carboxylic OH groups are grafted near to quantitative with PPG chains. In contrast, some aliphatic OH groups may remain unmodified after oxyalkylation. It is possible to distinguish between unreacted aliphatic OH groups and grafted aliphatic OH groups in the ^{31}P-NMR-spectrum. However, unmodified aliphatic OH groups will be acetylated as well in the acetylation process and would falsify the value of the statistical chain length. Therefore, the average chain length $n_{average}$ can be calculated since this value is only related to the PPG repetition unit.

$$n_{stat.} = \frac{I_{0.8-1.4\,ppm}\,(D)}{I_{1.8-2.1\,ppm}\,(C)} \qquad n_{average} = \frac{I_{0.8-1.4\,ppm}\,(D)}{I_{4.9-5.1\,ppm}\,(A)\cdot 3}$$

Calculation of theoretical yield and practical outcome of L-PO

The statistical or average chain length can be multiplied by the molecular weight of the PO repetition unit (M_{PO} = 58.08 g·mol^{-1}) to calculate the molecular weight of the grafted chains M_{chain}. The mass of the grafted PPG chain m_{chain} is obtained by multiplication of m_{chain} by the amount of substance of PB1000 n_{PB1000}. The theoretical yield of L-PO yield$_{theo.L-PO}$ is described by the sum of m_{chain} and m_{PB1000}. By dividing the amount of L-PO m_{L-PO} by the theoretical yield the practical outcome of L-PO can be determined. A correction of the OH number is possible by the division of n_{PB1000} by the theoretical yield of L-PO, followed by the calculation of the corrected lignin content in L-PO (lignin content $_{L-PO,corr.}$). The results are given in **Table S7** and **Table S8**.

$$\mathrm{M_{PPG\,chain}}[\mathrm{g \cdot mol^{-1}}] = \text{chain length } \cdot \mathrm{M_{PO}}[\mathrm{g \cdot mol^{-1}}]$$

$$\mathrm{m_{chain}}[\mathrm{g}] = \mathrm{n_{PB1000}}[\mathrm{mol}] \cdot \mathrm{M_{PPG\,chain}}\,[\mathrm{g \cdot mol^{-1}}]$$

$$\mathrm{n_{PB1000}}[\mathrm{mmol}] = \mathrm{m_{PB1000}}[\mathrm{g}] \cdot \mathrm{OH_{PB1000}}[\mathrm{mmol \cdot g^{-1}}]$$

$$\mathrm{yield_{theo.\ L-PO}}[\mathrm{g}] = \mathrm{m_{PB1000}}[\mathrm{g}] + \mathrm{m_{chain}}[\mathrm{g}]$$

$$\mathrm{yield_{L-PO}}\,[\%] = \frac{\mathrm{m_{L-PO}}\,[\mathrm{g}]}{\mathrm{yield_{theory}}[\mathrm{g}]} \cdot 100$$

$$\mathrm{OH_{corrected}}[\mathrm{mmol \cdot g^{-1}}] = \frac{\mathrm{n_{PB1000}}[\mathrm{mmol}]}{\mathrm{yield_{theory}}[\mathrm{g}]}$$

$$\text{lignin content}_{\mathrm{L-PO,corr.}}\,[\%] = \left(\frac{\mathrm{OH_{L-PO,corr.}}}{\mathrm{OH_{PB1000}}}\right) \cdot 100$$

Table S7: calculated parameter using statistical chain length.

	**L-PO 1***	**L-PO 2**	**L-PO 3**	**L-PO 4**
statistical chain length [-]	5.84	3.77	5.77	8.62
M_{chain} [g· mol^{-1}]	339.2	219.0	335.1	500.7
m_{chain} [g]	55.7	41.5	63.6	95.4
n_{PB1000} [mmol]	164.3	189.7	189.8	190.6
yield$_{theo}$ [g]	80.7	66.6	88.7	120.6
yield [%]	42	64	63	49
$OH_{corr.}$ [mmol· g^{-1}]	2.04	2.85	2.14	1.58
lignin content$_{corr.}$ [%]	31	38	28	21

Table S8: calculated parameter using average chain length.

	L-PO 1*	L-PO 2	L-PO 3	L-PO 4
average chain length [-]	6.79	4.86	7.53	10.86
M_{chain} [g· mol^{-}]	394.4	282.3	437.3	630.8
m_{chain} [g]	64.8	53.5	83.0	120.2
n_{PB1000} [mmol]	164.3	189.7	189.8	190.6
$yield_{theo}$ [g]	89.8	78.6	108.1	145.4
yield [%]	38	54	52	40
$OH_{corr.}$ [mmol· g^{-1}]	1.83	2.41	1.76	1.31
lignin $content_{corr.}$ [%]	28	32	23	17

Calculation of theoretical yield and practical outcome of L-PO-MAC (DS=100%)

The theoretical yield $m_{yield,theo.\ L\text{-}PO\text{-}MAC}$ is calculated by multiplication of the used molar ratio of $n_{L\text{-}PO}$ with the molecular weight of the methacrylate group $M_{methacryl\ group}$ followed by the addition of the used L-PO mass $m_{L\text{-}PO}$. This is the theoretical yield if 100 % of the available double bonds would be converted into reactive double bonds. This theoretical yield can be corrected by multiplication of the real OH conversion [%] determined by ^{31}P-NMR spectroscopy. Dividing the obtained yield of the product L-PO-MAC by the theoretical yield gives the yield of the synthesis.

$$m_{yield\ theo.\ L-PO-MAC} = n_{L-PO}[mol] \cdot M_{methacryl\ group}[g \cdot mol^{-1}] + m_{L-PO}[g]$$

$$n_{L-PO} = \frac{OH\ number\ [mmol \cdot g^{-1}] \cdot m_{L-PO}[g]}{1000}$$

$$yield_{L-PO-MAC}\ [\%] = \frac{m_{L-PO-MAC}}{m_{yield,theo,L-PO-MAC}} \cdot 100$$

$$M_{methacryl\ group} = 68.07\ g \cdot mol^{-1}$$

^{1}H-NMR-spectrum and assignment of methacrylated lignin L-PO-MAC

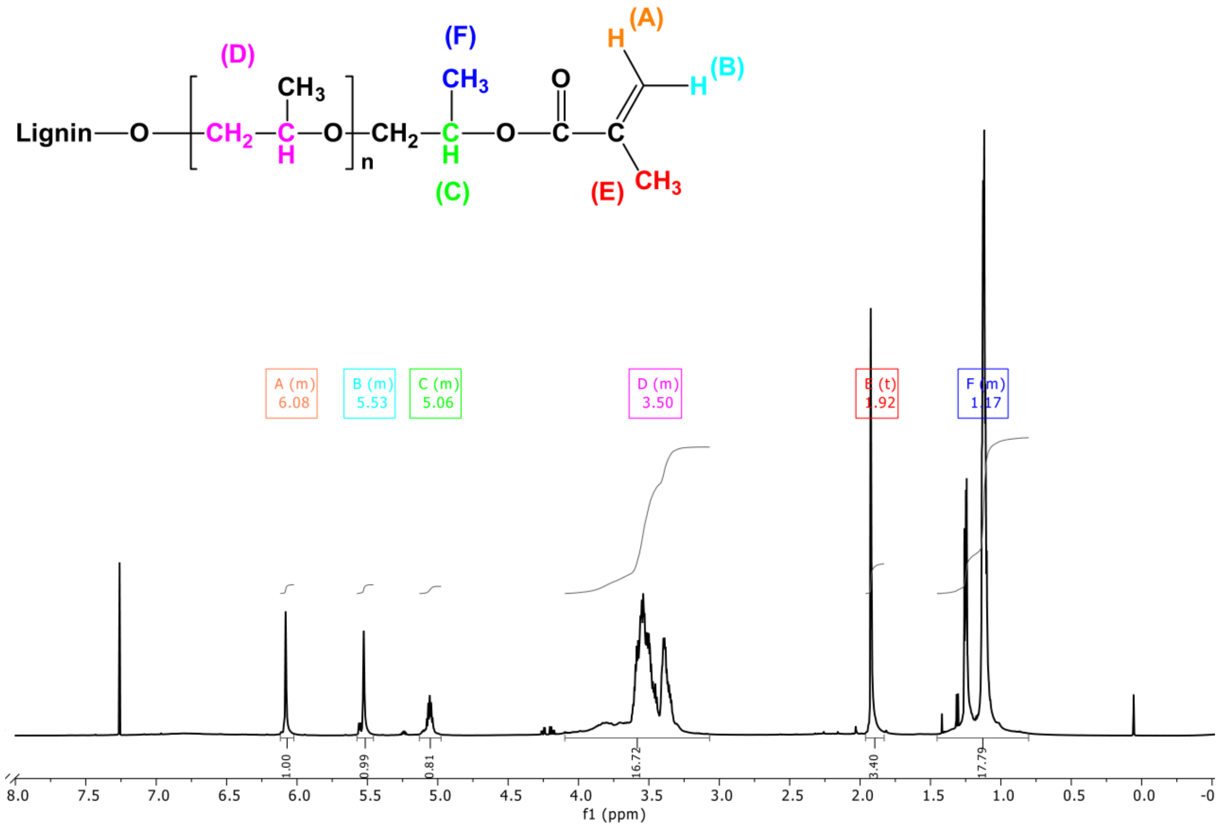

Figure S7: ^{1}H-NMR-spectrum of L-PO-MAC master mixture measured in $CDCl_3$ with assignments.

UV-Vis spectrum of L-PO-MAC

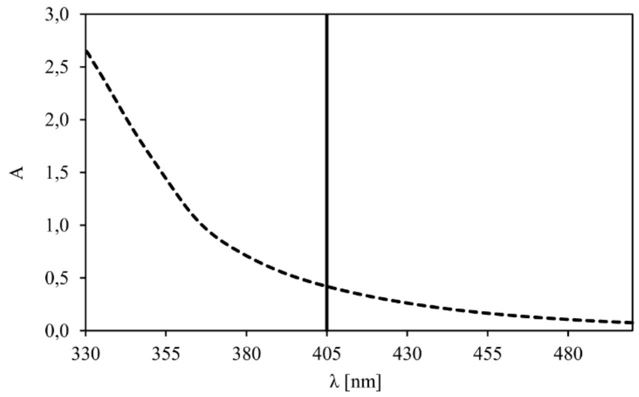

Figure S8: UV-VIS spectrum of L-PO-MAC master mixture (1 mg·mL^{-1}) Line at 405 nm describes the wavelength of the printer.

Gel permeation chromatography (GPC) data

GPC measurements were conducted according to the procedure described in the main manuscript. *OmniSEC Version 5.10.461* was used for the analysis of the chromatograms. First, a conventional calibration curve

was created from the measured polystyrene standards before result evaluation. For each chromatogram, baseline and peak limits were set manually.

Table S9: Calculated molar masses of lignin samples from GPC measurements.

**Sample[(a)]**	**M_n [g·mol^{-1}]**	**M_w [g·mol^{-1}]**	**M_w/M_n**
PB1000 (Ac)	906	2290	2.5
L-PO 1 (Ac)	2580	8435	3.3
L-PO 2 (Ac)	2549	9938	3.9
L-PO 3 (Ac)	3228	9947	3.1
L-PO 4 (Ac)	3396	12258	3.6
L-PO-MAC 1	3066	11679	3.8
L-PO-MAC 2	2673	11595	4.3
L-PO-MAC 3	3817	13136	3.4
L-PO-MAC 4	3712	14101	3.8
L-PO-MAC master mixture	3226	12177	3.8

[(a)] abbreviation: Ac, acetylated

Real time-NIR-photorheology data of L-PO-MAC formulations

Table S10: Formulations for real time-NIR-photorheology measurements.

**Formulation**	**L-PO-MAC [mg]**	**Ivocerin® [mg]**	**BAPO [mg]**
L-PO-MAC 4 + 0.6 mol% Ivocerin®	500	2.54	
L-PO-MAC 4 + 1.3 mol% Ivocerin®	500	5.04	
L-PO-MAC 4 + 2.5 mol% Ivocerin®	500	10.08	
L-PO-MAC 4 + 3.7 mol% Ivocerin®	500	15.03	
L-PO-MAC 4 + 6.2 mol% Ivocerin®	500	25.03	
L-PO-MAC 4 + 0.6 mol% BAPO	500		2.65
L-PO-MAC 4 + 1.3 mol% BAPO	500		5.26
L-PO-MAC 4 + 2.5 mol% BAPO	500		10.51
L-PO-MAC 4 + 3.7 mol% BAPO	500		15.69
L-PO-MAC 4 + 6.2 mol% BAPO	500		26.12
L-PO-MAC 1 + 2 wt% Ivocerin®	500	10	
L-PO-MAC 2 + 2 wt% Ivocerin®	500	10	
L-PO-MAC 3 + 2 wt% Ivocerin®	500	10	
L-PO-MAC master mixture + 2 wt% Ivocerin®	500	10	

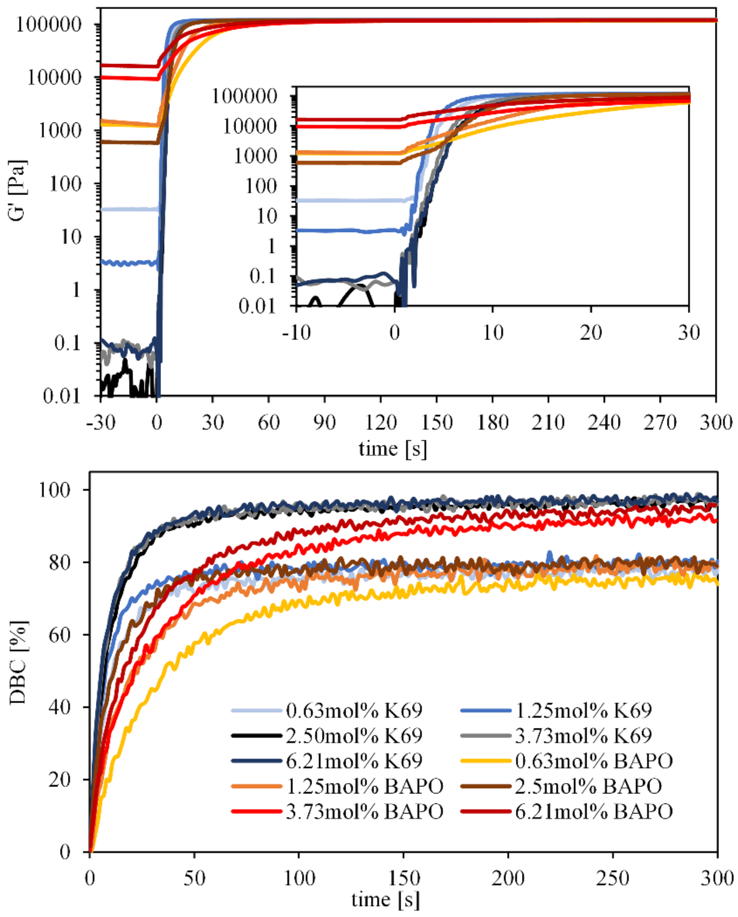

Figure S9: Real time-NIR-photorheology results of L-PO-MAC 4 formulations with different amounts of photoinitiators: storage modulus G' (top) and double bond conversion DBC (bottom) curves. The irradiation of the samples starts at t = 0 s. For a better visibility, the G' curves are also enlarged in the time interval from -10 to 30 s.

Table S11: Photorheology data of L-PO-MAC 4 formulations with different amounts of photoinitiators.

Formulation	t_g [s]	G'_{max} [kPa]	DBC at t_g [%]	DBC_{end} [%]
0.6 mol% Ivocerin®	2.8 ± 0.1	121.9 ± 2.7	19.5 ± 1.2	77.8 ± 0.6
1.3 mol% Ivocerin®	2.4 ± 0.0	120.2 ± 0.5	21.1 ± 1.6	80.1 ± 0.5
2.5 mol% Ivocerin®	4.2 ± 0.2	118.0 ± 1.9	38.1 ± 0.2	96.5 ± 0.3
3.7 mol% Ivocerin®	3.5 ± 0.3	115.9 ± 1.6	35.9 ± 1.2	96.1 ± 0.0
6.2 mol% Ivocerin®	3.9 ± 0.0	118.3 ± 0.4	41.1 ± 1.9	97.2 ± 0.4
0.6 mol% BAPO	4.2 ± 0.8	116.6 ± 1.7	10.3 ± 2.7	75.5 ± 0.5
1.3 mol% BAPO	4.2 ± 0.0	119.6 ± 1.2	18.6 ± 0.1	78.9 ± 0.6
2.5 mol% BAPO	4.9 ± 0.5	120.4 ± 1.2	33.8 ± 1.2	79.7 ± 0.7
3.7 mol% BAPO	5.2 ± 0.8	117.8 ± 1.3	22.8 ± 2.4	92.1 ± 0.6
6.2 mol% BAPO	4.2 ± 0.0	118.9 ± 0.4	20.8 ± 1.4	94.9 ± 0.6

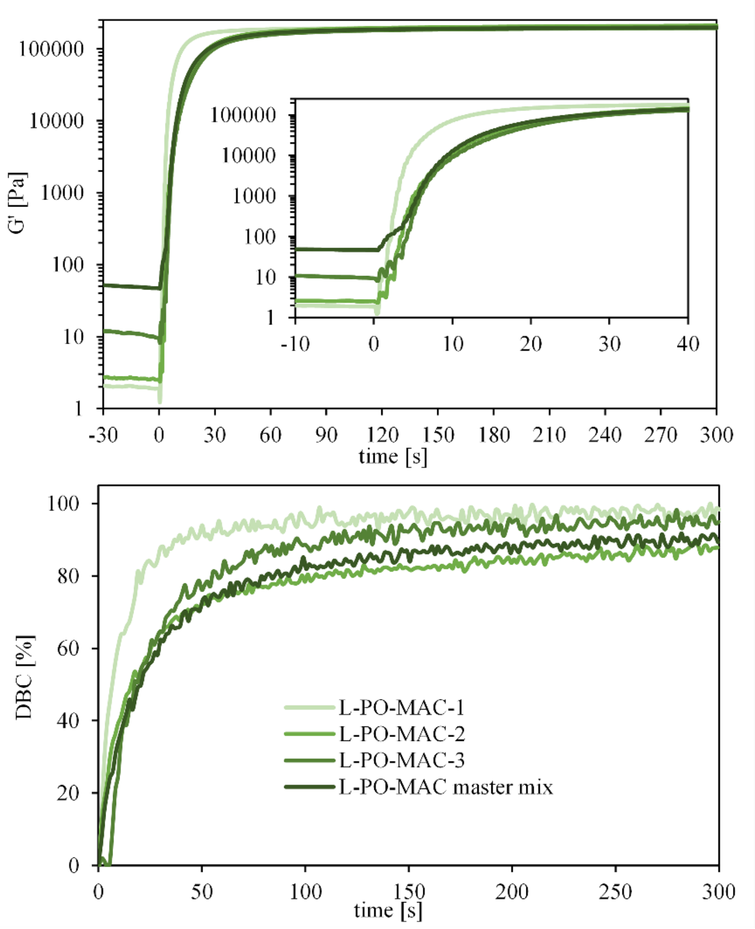

Figure S10: Real time-NIR-photorheology results of L-PO-MAC 1-3 and master mixture formulations with 2 wt% of Ivocerin® as photoinitiator: storage modulus G' (top) and double bond conversion DBC (bottom) curves. The irradiation of the samples starts at t = 0 s. For a better visibility, the G' curves are also enlarged in the time interval from -10 to 40 s.

Table S12: Photorheology data of L-PO-MAC 1-3 and master mixture formulations.

Formulation	t_g [s]	G'_{max} [kPa]	DBC at t_g [%]	DBC_{end} [%]
L-PO-MAC 1	2.7 ± 0.3	198.9 ± 1.8	27.6 ± 3.0	97.5 ± 0.6
L-PO-MAC 2	5.0 ± 1.1	209.0 ± 4.1	26.8 ± 2.9	87.3 ± 2.4
L-PO-MAC 3	5.2 ± 0.6	194.3 ± 3.2	33.4 ± 0.2	95.4 ± 1.0
L-PO-MAC master mixture	4.8 ± 0.7	197.0 ± 4.1	20.2 ± 3.9	90.1 ± 2.3

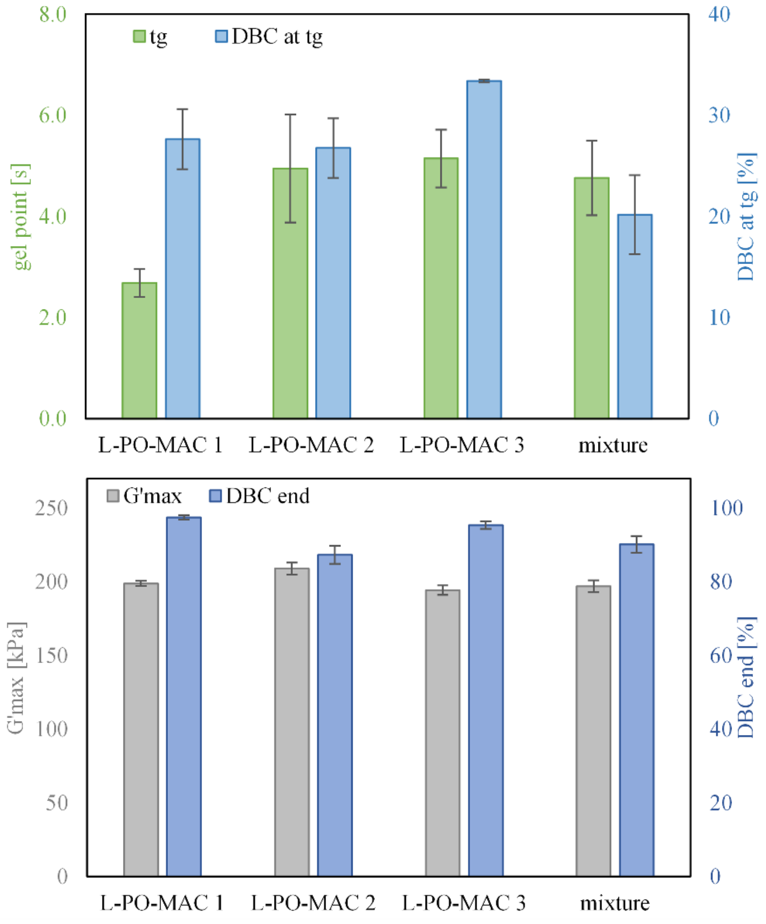

Figure S11: Real time-NIR-photorheology results of L-PO-MAC 1-3 and L-PO-MAC master mixture formulations with 2wt% of Ivocerin®: gel point t_g and double bond conversion DBC at gel point t_g (top). Maximum storage modulus G'_{max} and conversion DBC_{end} (bottom).

3D printing and characterization of the printed specimen

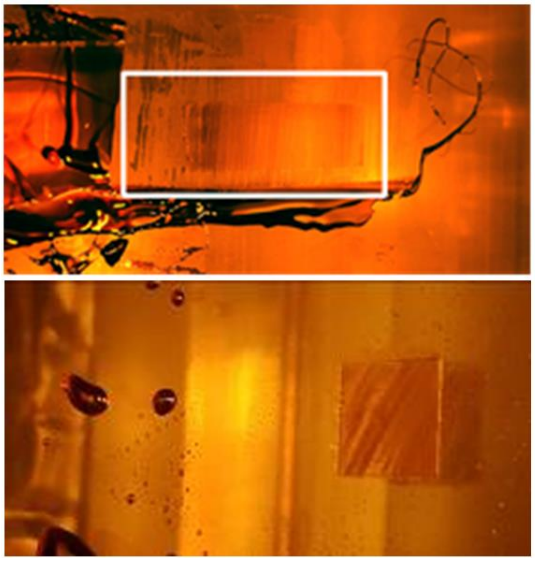

Figure S12: Exposure tests with L-PO-MAC master mixture. Formation of an undetachable thin film (top, highlighted by white frame) and platelet formation after optimizing the printing parameters (bottom).

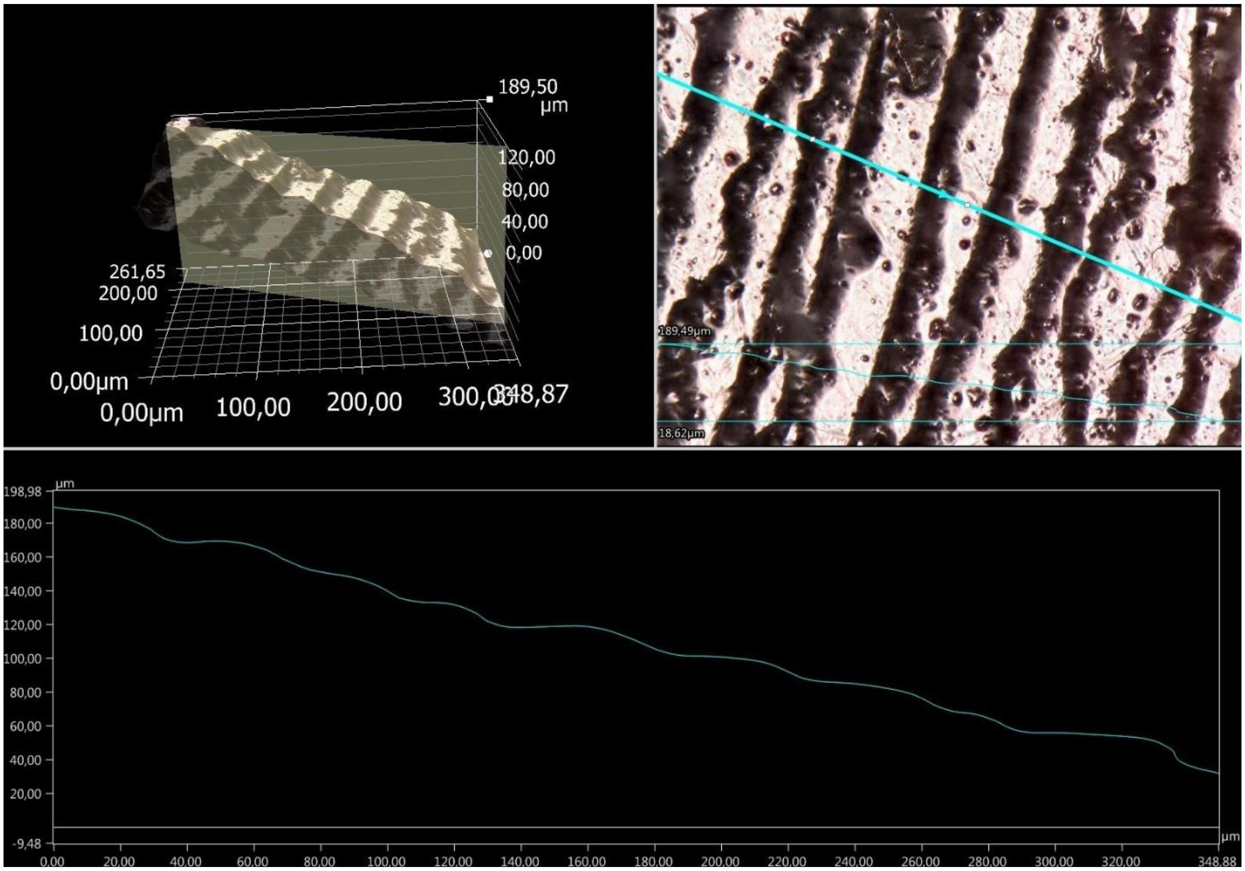

Figure S13: Digital microscopy analysis of 3D printed lignin specimen. Measuring range of the specimen (top) and height profile of the analyzed area (bottom). Since the measurement was performed after SEM analysis, the surface of the 3D printed specimen is golden.

References

(1) del Río, J. C.; Rencoret, J.; Prinsen, P.; Martínez, Á. T.; Ralph, J.; Gutiérrez, A. Structural Characterization of Wheat Straw Lignin as Revealed by Analytical Pyrolysis, 2D-NMR, and Reductive Cleavage Methods. *J. Agric. Food Chem.* **2012**, *60* (23), 5922–5935. https://doi.org/10.1021/jf301002n.

(2) Sun, R.; Lawther, J. M.; Banks, W. B. Effects of Extraction Time and Different Alkalis on the Composition of Alkali-Soluble Wheat Straw Lignins. *J. Agric. Food Chem.* **1996**, *44* (12), 3965–3970. https://doi.org/10.1021/jf9600566.

(3) Wen, J.-L.; Xue, B.-L.; Xu, F.; Sun, R.-C. Unveiling the Structural Heterogeneity of Bamboo Lignin by In Situ HSQC NMR Technique. *BioEnergy Res.* **2012**, *5* (4), 886–903. https://doi.org/10.1007/s12155-012-9203-5.

(4) Wen, J.-L.; Sun, S.-L.; Xue, B.-L.; Sun, R.-C. Recent Advances in Characterization of Lignin Polymer by Solution-State Nuclear Magnetic Resonance (NMR) Methodology. *Materials* **2013**, *6* (1), 359–391. https://doi.org/10.3390/ma6010359.

(5) Yuan, T.-Q.; Sun, S.-N.; Xu, F.; Sun, R.-C. Characterization of Lignin Structures and Lignin–Carbohydrate Complex (LCC) Linkages by Quantitative ^{13}C and 2D HSQC NMR Spectroscopy. *J. Agric. Food Chem.* **2011**, *59* (19), 10604–10614. https://doi.org/10.1021/jf2031549.

(6) Ahvazi, B.; Wojciechowicz, O.; Ton-That, T.-M.; Hawari, J. Preparation of Lignopolyols from Wheat Straw Soda Lignin. *J. Agric. Food Chem.* **2011**, *59* (19), 10505–10516. https://doi.org/10.1021/jf202452m.
(7) Balakshin, M.; Capanema, E. On the Quantification of Lignin Hydroxyl Groups With 31P and 13C NMR Spectroscopy. *J. Wood Chem. Technol.* **2015**, *35* (3), 220–237. https://doi.org/10.1080/02773813.2014.928328.
